# Mucin adsorbed by *E. coli* can affect neutrophil activation *in vitro*

**DOI:** 10.1002/2211-5463.12770

**Published:** 2019-12-19

**Authors:** Elena Mikhalchik, Nadezhda Balabushevich, Tatiana Vakhrusheva, Alexey Sokolov, Julia Baykova, Daria Rakitina, Petr Scherbakov, Sergey Gusev, Alexander Gusev, Zaira Kharaeva, Olga Bukato, Olga Pobeguts

**Affiliations:** ^1^ Federal Research and Clinical Center of Physical‐Chemical Medicine Moscow Russia; ^2^ Department of Chemistry Lomonosov Moscow State University Russia; ^3^ Institute of Experimental Medicine St. Petersburg Russia; ^4^ Kabardino‐Balkarian State University Nalchik Russia

**Keywords:** *E. coli*, mucin, neutrophils, outer membrane proteins, plasma proteins, reactive oxygen species

## Abstract

Bacteria colonizing human intestine adhere to the gut mucosa and avoid the innate immune system. We previously demonstrated that *Escherichia coli* isolates can adsorb mucin from a diluted solution *in vitro*. Here, we evaluated the effect of mucin adsorption by *E. coli* cells on neutrophil activation *in vitro*. Activation was evaluated based on the detection of reactive oxygen species production by a chemiluminescent reaction (ChL), observation of morphological alterations in neutrophils and detection of exocytosis of myeloperoxidase and lactoferrin. We report that mucin adsorbed by cells of SharL1 isolate from Crohn's disease patient's inflamed ileum suppressed the potential for the activation of neutrophils in whole blood. Also, the binding of plasma complement proteins and immunoglobulins to the bacteria was reduced. Desialylated mucin, despite having the same adsorption efficiency to bacteria, had no effect on the blood ChL response. The effect of mucin suggests that it shields epitopes that interact with neutrophils and plasma proteins on the bacterial outer membrane. Potential candidates for these epitopes were identified among the proteins within the bacterial outer membrane fraction by 2D‐PAGE, fluorescent mucin binding on a blot and HPLC‐MS/MS. *In vitro*, the following proteins demonstrated mucin adsorption: outer membrane porins (OmpA, OmpC, OmpD and OmpF), adhesin OmpX, the membrane assembly factor OmpW, cobalamine transporter, ferrum uptake protein and the elongation factor Ef Tu‐1. In addition to their other functions, these proteins are known to be bacterial surface antigens. Therefore, the shielding of epitopes by mucin may affect the dynamics and intensity of an immune response.

## Graphical abstract

*Escherichia coli* isolates from healthy and inflamed intestines of humans adsorbed mucin from solution. Mucin adsorbed by cells of SharL1 isolate from Crohn's disease patient suppressed their potential for neutrophil activation and their ability to bind plasma complement proteins and immunoglobulins. OmpA, OmpC, OmpD, OmpF, OmpX, OmpW, cobalamine transporter, ferrum uptake protein and the Ef Tu‐1 bound mucin *in vitro*.

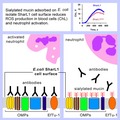

**Abbreviations**
2D‐PAGE: two‐dimensional polyacrylamide gel electrophoresis
ChL: chemiluminescent reaction
DW: dry weight
emPAI: exponentially modified protein abundance index
HPLC‐MS/MS: high‐pressure liquid chromatography with tandem mass spectrometry
LF: lactoferrin
MPO: myeloperoxidase
OMP: outer membrane protein
ROS: reactive oxygen species

For the successful colonization of the human intestine, bacteria have to adhere to the gut mucosa to avoid an attack from the innate immune system – complement system and neutrophils.

The interaction of bacteria with the major mucus component glycoprotein mucin plays an important but an ambiguous role in this process. On one side, bacteria use specific proteases to disrupt the polymerized mucin layer that protects the intestinal epithelium (see for review Refs 1, 2). Mucin degradation was observed as a result of the activity of individual organisms (bacteria, parasitic protozoa and helminths) or whole microbial communities. In most cases, the first step of the process involves sulfatases, sialases and glycosidases, which desulfate and degrade mucin oligosaccharides, exposing the peptide backbone for serine and cysteine protease attack 2. Another mechanism was described for proteases (identified in *Schigella* and *Escherichia coli*) that used O‐glycosylated mucin as a substrate 3. In all cases, proteolysis targeting the N‐ and C‐polymerizing ends of mucin leads to the disassembly of mucin macromolecules, which decreases mucus viscosity and protective functions. Mucin monomers and smaller cleavage products are produced in these reactions 2.

On the other hand, bacteria (including *E. coli*) were reported to attach to the mucin layer through fimbrial [type 1 pili (fimbriae)] and afimbrial (multivalent adhesion molecule) adhesins 4, 5 as well as induce mucin hypersecretion in the rabbits' ileal loop and cultured human mucin‐secreting intestinal HT29‐MTX cells 6. *E. coli* was also reported to attach to the intestinal mucus via its flagellum 7.

After permeation through the mucosal barrier, bacteria can trigger innate immune response sensors (Peyer's glands and goblet cells; see 8 for review) and induce an inflammatory reaction. Additionally, bacteria can make contacts with gut luminal neutrophils without mucosal invasion, since many inflammatory conditions are marked by the recruitment of neutrophils to the intestinal lumen 9, 10. However, the role of mucin in the activation of innate immune factors by bacteria is still unknown.

The majority of researchers study the adhesion of bacteria to polymerized mucin (for review see Ref. 1). At the same time, the gut contents contain a great amount of free soluble mucin that is produced by constant secretion 11 and exfoliation during the renewal of the mucus layer and mucin degradation by anaerobic microflora 12. However, the possible roles that soluble mucin could play in bacterial–host interaction have been much less frequently examined.

On the host side, polymerized and soluble mucin could have a direct effect on neutrophils. Mucin treatment of plastic surfaces inhibited neutrophil adhesion and activation, and the effect was the most pronounced in the presence of plasma proteins, mainly the complement system 13. In contrast, in the absence of plasma proteins, mucins from the healthy human eye mucosa activated neutrophils *in vitro*, and some pathologies abolished this effect 14.

The aim of the current study was to evaluate the effect of mucin adsorption by *E. coli* cells on neutrophil activation *in vitro*. We previously demonstrated that *E. coli* isolates from the healthy or inflamed (Crohn's disease) human intestine and the laboratory strain DH5α were able to adsorb mucin from a diluted solution *in vitro* 15. Among our findings, the amount of adsorbed mucin varied greatly between *E. coli* isolates (more than threefold). No significant difference in the ability to adsorb mucin was observed between the isolates from the inflamed and healthy intestine and the laboratory strain 15.

In the current study, we have compared the influence of mucin on strains with a similar ability to adsorb mucin – the clinical isolate SharL1 and the laboratory strain DH5α. Neutrophil activation by bacteria *in vitro* was analysed by measuring the production of reactive oxygen species (ROS) in whole blood or in neutrophil suspension, myeloperoxidase (MPO) and lactoferrin (LF) exocytosis in blood, neutrophils' morphological alterations and changes in plasma protein contents. Bacterial outer membrane proteins that adsorb mucin *in vitro* were identified by HPLC–mass‐spectrometry identification.

## Methods

### *Escherichia coli* strain isolation, cultivation and characterization

*Escherichia coli* were isolated as described in File S1 from Crohn's patients (*n* = 5, four male and one female; age, 23–47; median, 33), one patient without endoscopically confirmed inflammatory bowel disease (IBD) (male, age 14), healthy faeces donors (*n* = 4, three male and one female; age, 19–28; median, 19.5) and healthy blood donors (*n* = 5, female; age, 21–64; median, 38) who were enrolled in the study and gave written informed consent. The study methodologies were conformed to the standards set by the Declaration of Helsinki and were approved by the local Ethics Committee. The clinical and endoscopic activities of the disease were evaluated according to Refs 16, 17 and are shown in File S2.

*Escherichia coli* isolation was performed as follows. The liquid ileal contents or faeces were diluted 10^3^‐ to 10^6^‐fold, plated onto agar LB medium and incubated at 37 °C for 16 h. Individual colony species were identified by a Biotyper system with a Bruker Microflex mass spectrometer (Bruker, Bremen, Germany).

For further assays, bacteria were cultivated under aerobic conditions in LB at 37 °C (200 r.p.m.) for 14 h. The cells were harvested by centrifugation (3500 ***g***, 15 min), and the pellet was washed twice with PBS. The bacterial suspensions were normalized by absorbance at 540 nm (OD 540). 1 OD unit in a 1‐mL cuvette was equal to 0.2 mg·mL^−1^ of bacterial dry weight (DW) and contained 8∙10^8^ CFU·mL^−1^.

The SharL1 *E. coli* isolate was isolated from the ileum of Crohn's disease patient (male, 26 years old). The patient's detailed diagnosis is as follows: Crohn's disease, ileocolitis, ileum, sigmoid and caecum affected; lymphofollicular hyperplasia of the ileum, ileitis and aphthosic proctosigmoiditis. SharL1 was isolated after a diagnostic endoscopy (Moscow Clinical Research Centre of Gastroenterology). Material collection was approved by the local Ethics Committee, as the patient had provided written informed consent for research and data publication.

Phylogroup determination of SharL1 was performed by PCR with primers for the *tsp*, *yja* and *chuA* genes as described in Ref. 18.

The adhesion ability of the SharL1 isolate was tested on a monolayer of CaCo cells. The isolate was cultivated on LB (37 °C, 200 r.p.m.) and harvested by centrifugation (3500 ***g***, 15 min) at mid‐log phase. The pellet was washed with PBS and then resuspended in PBS. Next, 150 µL of bacterial suspension (OD 620 = 0.2) was mixed with 5 mL DMEM + 20% FBS. Bacteria in DMEM (500 µL) were added to the CaCo cells, grown in a 24‐well plate until monolayer formation (approximately 500 000 CaCo cells per well). The bacterial suspension (control 1) was collected at this point and plated onto LB‐agar plates at 10^−3^‐ and 10^−4^‐fold dilutions. The plate containing the CaCo cells and bacteria was incubated for 3 h at 37 °C. For the invasion test, the cell suspension was treated with 1 mL of DMEM (20% FBS) containing 300 mg·L^−1^ of gentamicin for 1 h at 37 °C. After incubation, the monolayer was gently washed twice with PBS to remove any unbound bacteria; the cells were removed from the plate by trypsin (200 µL) and lysed with 400 µL of 0.5% Triton X‐100 in DMEM (20% FBS). The solution was mixed and plated on agar LB at 10^−2^‐ and 10^−3^‐fold dilutions (5 µL per agar plate).

Adhesion + invasion values were determined as follows: (a) the number of bacteria adhered‐invaded per CaCo cell = CFU per agar plate after adhesion × dilution/500 000 (the number of CaCo cells per well) and (b) the % of adhered‐invaded bacteria in the bacterial suspension = CFU per agar plate after adhesion/CFU per agar plate in control 1 × 100 (considering corresponding dilutions).

The invasion value was determined as a % of the adhered‐invaded bacteria that survived gentamicin treatment.

The bacterial mobility of the SharL1 isolate was determined by a needle stuck into the thick layer of 0.4% LB agar. After 24 h of incubation at 37 °C, the diameter of the bacterial spot was measured.

### Mucin purification and modification

In all experiments, we used naturally derived type III mucin from porcine stomach with bound 0.5–1.5% sialic acid (m1778) (Sigma‐Aldrich, St. Louis, MO, USA), which is structurally related to human gastric mucin 19. The approaches for its purification and quantification were described previously 20.

The mucin solution (1–5 mg·mL^−1^) was purified using gel filtration on a Sephadex G‐200 (Bio‐Logic LP System; Bio‐Rad, Hercules, CA, USA) with detection at 214, 260 and 480 nm and a specific assay performed with Schiff's method; the fractions containing mucin were combined and freeze‐dried (native mucin). Fluorescein isothiocyanate‐labelled mucin (FITC‐mucin) (17 ± 2% modified amino groups) and desialylated mucin (82 ± 4% deleted sialic acids) were prepared and purified using gel filtration as described previously 20.

### Mucin adsorption by bacteria

Bacteria (0.09 mg DW) were incubated in 1 mL of 0.15 m NaCl containing 0.1 mg·mL^−1^ mucin (or without mucin) at 25 °C for 1 h. After centrifugation (20 min, 900 ***g***), the mucin concentration in the supernatants was assessed by absorption at 214 nm (*A*_214_), with 0.1 mg·mL^−1^ mucin solution used as a reference sample 15. From the difference in these concentrations, the amount of adsorbed mucin was calculated. The cell pellets were resuspended in 0.15 m NaCl for further analyses. The mucin‐treated cells were marked as SharL1^muc^ and DH5α^muc^.

### Blood sampling and neutrophils isolation

Blood (5 mL) was collected by venepuncture using sodium citrate vacutainers. Material collection was approved by the local Ethics Committee, and donors provided written informed consent for research and data publication. The experiments were performed within 4 h after blood sampling. To isolate neutrophils, the blood was immediately layered upon a 1.077/1.119 double Histopaque gradient and centrifuged for 40 min at 400 ***g***. The neutrophils were washed twice with the Krebs‐Ringer solution (10 min, 400 ***g***) and resuspended (25 × 10^6^ cell·mL^−1^) in autologous blood plasma (Nph + plasma) or in Krebs‐Ringer buffer solution (Nph).

### Chemiluminescent assay for ROS production

Reactive oxygen species generation was used as a marker of leucocyte activation. The chemiluminescent assay was a simple and sensitive method to determine ROS in a neutrophil suspension and diluted blood 21. A Luminometer 1200 (DiSoft, Moscow, Russia) was applied to measure chemiluminescence (ChL) in two probes simultaneously under mild stirring at 37 °C with continuous data recording. The blood samples or neutrophil suspensions were added to Krebs‐Ringer buffer solution with 0.3 mm luminol (pH 7.4). The final blood dilution was 1 : 25 and the isolated neutrophil concentration was 5 × 10^5^ cells·mL^−1^, with the total probe volume equal to 500 µL. The spontaneous ChL was measured for 3 min; then, 20 µL of the bacterial suspension was added (4 µg·mL^−1^ DW) and the ChL kinetics and response maxima were registered. To study the effects of mucin on the ChL response of diluted blood, 20 µL of 30 µg·mL^−1^ mucin solution or 0.15 m NaCl was added before the bacterial suspension.

### Co‐incubation of whole blood with bacteria

To simultaneously evaluate the effects of adsorbed mucin on ROS generation by leucocytes in blood, neutrophil morphology and blood plasma proteins, the blood samples were incubated with bacterial suspensions or reference solutions. Then, 400 µL of blood was mixed with 100 µL of SharL1 or SharL1^muc^ suspension (final concentration of 30 µg·mL^−1^ DW), 100 µL of 0.15 m NaCl or 100 µL of 0.1 mg·mL^−1^ mucin in 0.15 m NaCl. Blood smears were immediately prepared (2 min time points), and the other probes were incubated at 25 °C for 5 and 20 min. After incubation, the blood smears were prepared for morphological analysis and 20 µL aliquots were immediately used to measure the initial ChL value (2 s after sample addition). Then, the rest of the samples were centrifuged (10 min, 400 ***g***) and the plasma was separated and frozen at −80 °C for MPO, LF and other plasma protein assays.

### Analysis of neutrophil morphology

Blood cells were evaluated by light microscopy in blood smears 22. The blood smears were stained according to the Romanovskii–Giemsa technique and then analysed using a light Motic BA223 microscope (Motic, Kowloon, Hong Kong) equipped with a 3CCD KYF32 digital camera. Image processing was performed with a MECOS‐C image analysis system (MECOS, Moscow, Russia) in semiautomatic mode. No less than 50 neutrophils were analysed in each smear for morphologic signs of neutrophil activation after incubation with bacteria, including the number of vacuoles formed 23.

### Measurement of myeloperoxidase and lactoferrin concentrations

To determine the MPO and LF concentrations in blood plasma after incubation of whole blood with bacteria for 5 min, an ELISA was performed as described previously 24, 25.

### Isolation of *E. coli* cell membranes

The isolation of cell membrane proteins was performed with the carbonate method 26. The bacterial culture in a mid‐log phase was harvested by centrifugation for 8 min at 2500 ***g***. The pellet was washed once with PBS and resuspended in 200 µL of Tris/HCl buffer pH 7.5 with 1 µL of a nuclease mixture (Promega, Madison, WI, USA). The cells were lysed by ultrasonication for 1 min and centrifuged for 8 min at 2500 ***g***. The supernatant was added to 1.5 mL of 100 mm Na_2_CO_3_ and incubated on ice with shaking for 1 h. The membrane proteins' fraction was precipitated by ultracentrifugation at 115 000 ***g*** for 30 min, and the pellet was washed with 50 mm Tris/HCl buffer pH 7.5 and collected again by centrifugation using the same conditions.

### Mucin‐binding assay for *E. coli* membrane proteins

The membrane proteins from the *E. coli* SharL1 isolate were separated by 2D‐SDS/PAGE (2D electrophoresis protocol in Ref. 27) and transferred to a nitrocellulose filter Hybond‐P with a Bio‐Rad MiniCell according to the manufacturer's protocol. The active binding groups on the filter were blocked by incubation for 1 h at 25 °C in 3% BSA solution in PBS containing 300 mm NaCl. Adsorption of the fluorescein isothiocyanate‐labelled mucin was performed by incubation at 25 °C for 4 h in binding buffer (5 mg·mL^−1^ FITC‐mucin, 300 mm NaCl, 3 mm MgCl_2_, 1 mm DTT and 0.05% Tween‐20). The filters were washed three times for 5 min each in PBS and scanned on a fluorescent scanner Typhoon by GE Healthcare (Chicago, IL, USA) (Blue laser, 520‐nm bandpass filter, scanning parameters: pixel size 50 µm, PMT voltage 550). Mucin‐adsorbing protein spots were excised from the filter and subjected to trypsinolysis for consequent protein analysis by HPLC‐MS/MS.

### Protein trypsinolysis on nitrocellulose filter

The nitrocellulose filter fragments were covered with 100 mm NH_4_HCO_3_ containing 0.1% sodium deoxycholate (DCNa), incubated in an ultrasonication bath for 10 min and heated for 5 min at 95 °C. After cooling the samples, 5 mm of the reducing agent tris(2‐carboxyethyl)phosphine (TCEP) was added and the samples were incubated at 37 °C for 1 h. Then, 30 mm iodoacetamide (IAA) was added and the samples were maintained at room temperature (RT) for 30 min. To avoid chemical modifications and remove the unreacted IAA, the samples were treated with 2.5 mm TCEP and incubated at RT for 30 min. Protein hydrolysis was performed with trypsin (20 µg per sample, Trypsin Gold, Mass Spectrometry Grade; Promega) for 16 h at 37 °C. After that, an equal volume of ethyl acetate was added to the sample and its pH was adjusted to 2.0 with trichloroacetic acid (TCA). At this point, trypsinolysis stopped and DCNa was precipitated and moved to the organic phase. After 5 min of intensive shaking and 5 min of centrifugation at 16 000 ***g*** at 20 °C, the lower water phase was collected and cleaned with C18 cartridges [Discovery DSC‐18 Tube (Supelco; Sigma‐Aldrich)] according to the manufacturer's protocol. The achieved peptide extracts were dried in a SpeedVac (Labconco, Kansas City, MO, USA) and dissolved in 15 µL of HPLC‐MS/MS sample buffer containing 3% acetonitrile and 0.1% trifluoroacetic acid.

### Trypsinolysis of plasma samples

After 5 min of incubation (10 µL), plasma samples from whole blood were diluted 50‐fold and proteins were precipitated with 10% TCA. After incubation at −20 °C for 1 h, the samples were centrifuged for 15 min at 16 000 ***g***. The pellet was washed twice with cold acetone, dried, diluted in Laemmli's buffer and heated at 95 °C for 5 min. After centrifugation for 5 min at 16 000 ***g***, the protein concentration was measured in the supernatant by Bradford assay (Bio‐Rad Quick Start Bradford Protein Assay, Hercules, CA, USA). The samples (50 µg of protein per lane) were loaded and separated by 7.5% SDS/PAGE. The gel was stained with Coomassie G‐250 and destained with 10% acetic acid. The lanes were divided into three parts, and the major albumin protein spot was cut out to increase the number of identified proteins. Trypsinolysis of proteins in the gel was performed as described in Ref. 28. The peptides' extracts were dried in a SpeedVac (Labconco) and dissolved in 15 µL of LC‐MS/MS sample buffer containing 3% acetonitrile and 0.1% trifluoroacetic acid.

### HPLC‐MS/MS analysis

The LC‐MS/MS analysis of the tryptic peptides was performed using an Ultimate‐3000 HPLC system (Thermo Scientific, Waltham, MA, USA) coupled to a maXis qTOF after the HDC‐cell upgrade (Bruker) with a nanoelectrospray source. The chromatographic separation of the peptides was performed on a C‐18 reverse phase column (Zorbax 300SB‐C18, 150 mm × 75 µm, particle diameter 3.5 µm; Agilent, Santa Clara, CA, USA). The gradient parameters were as follows: 5–35% acetonitrile in aqueous 0.1% (v/v) formic acid and a column flow rate of 0.3 µL·min^−1^. The gradient duration was 60 min (plasma samples) or 5 min (proteins from the nitrocellulose filters). Positive MS and MS/MS spectra were acquired using autoMS/MS mode (capillary voltage, 1700; the curtain gas flow is 4 L and the temperature is 170 °C, spectra rate 4 Hz, 20 precursors; *m/z* range, 200–1500; and active exclusion after two spectra and release after 0.5 min). The lists of compounds (mgf files) were generated after a lock mass calibration with a Compass DataAnalysis (Bruker). If one sample consisted of several injections, all generated compound lists were united into one.

### Protein identification and quantitative analysis

Protein identification was performed by peptide search with the Mascot Data Search with the following parameters: peptide mass tolerance, 0.05 Da; fragment mass tolerance, 0.1 Da; variable modifications such as carbamidomethyl (C) and oxidation (M); for the samples from PAGE, propionamide (C) and oxidation (M); cutting enzyme trypsin; and 1 missed cleavage per peptide was allowed. The peptide search for protein identification was performed as follows. The plasma samples were compared to the SwissProt database (taxonomy Homo_sapiens). The SharL1 samples versus the protein database (peptides) samples from the *E. coli* strain Nissle 1917 focused on a gut‐colonizing strain from the B2 phylogroup, which is the same phylogroup as SharL1 (File S1). A protein was considered as identified by no less than two unique peptides with a score above the threshold.

The protein abundances were evaluated by a label‐free method using the emPAI (Exponentially Modified Protein Abundance Index) determined by the Mascot Data Search for each identified protein 29. For plasma samples, the median values of the emPAI indexes were calculated for two biological repeats. The emPAI of each protein was normalized to the total emPAI of the sample. For the identification of proteins on nitrocellulose filters after 2D‐PAGE, the major protein in each spot was identified as having the maximum emPAI coefficient.

### Statistical data analysis

The statistical analysis was performed using statistica 6.0 (TIBCO Software Inc, Palo Alto, CA, USA). The results are presented as medium values (*n* = 3–5) ± standard deviation or medians. The values were compared using Student's *t*‐test or paired *t*‐test for independent samples. The difference was considered significant at a *P*‐value < 0.05.

## Results

### SharL1 isolate characteristics

Phylogroup typing of the SharL1 isolate by PCR assay resulted in PCR products for all three genes tested (File S1), indicating that the SharL1 isolate belongs to phylogroup B2 [according to Ref. 18.

Adhesion and invasion comparative data for the SharL1 isolate and MG1655 laboratory strain are given in Table 1. According to these data, the SharL1 demonstrates 24 times more effective adhesion to CaCo cells than MG1655, which can be defined as adhesive. At the same time, both SharL1 and MG1655 demonstrated no invasion activity.

**Table 1:** Adhesion and invasion characteristics of the SharL1 isolate and laboratory strain MG1655. Values are averaged from three biological and three mechanical repeats (CFU, median value ± standard deviation).

	SharL1	MG1655
Control 1, ×10^4^	12.6 ± 3.9	18.9 ± 2.5
Adhesion + invasion, ×10^3^	30.1 ± 4.6	2 ± 0.2
% of bacteria that adhered (invaded) to CaCo cells	2.4 ± 1.2	0.1 ± 0.1
Invasion	0	0

The mobility demonstrated by the SharL1 isolate (17.54 ± 7.47 mm) was five times higher than the laboratory strain MG1655 (3.46 ± 0.26 mm).

### Kinetics of ROS production by blood cells

**Figure 1:**
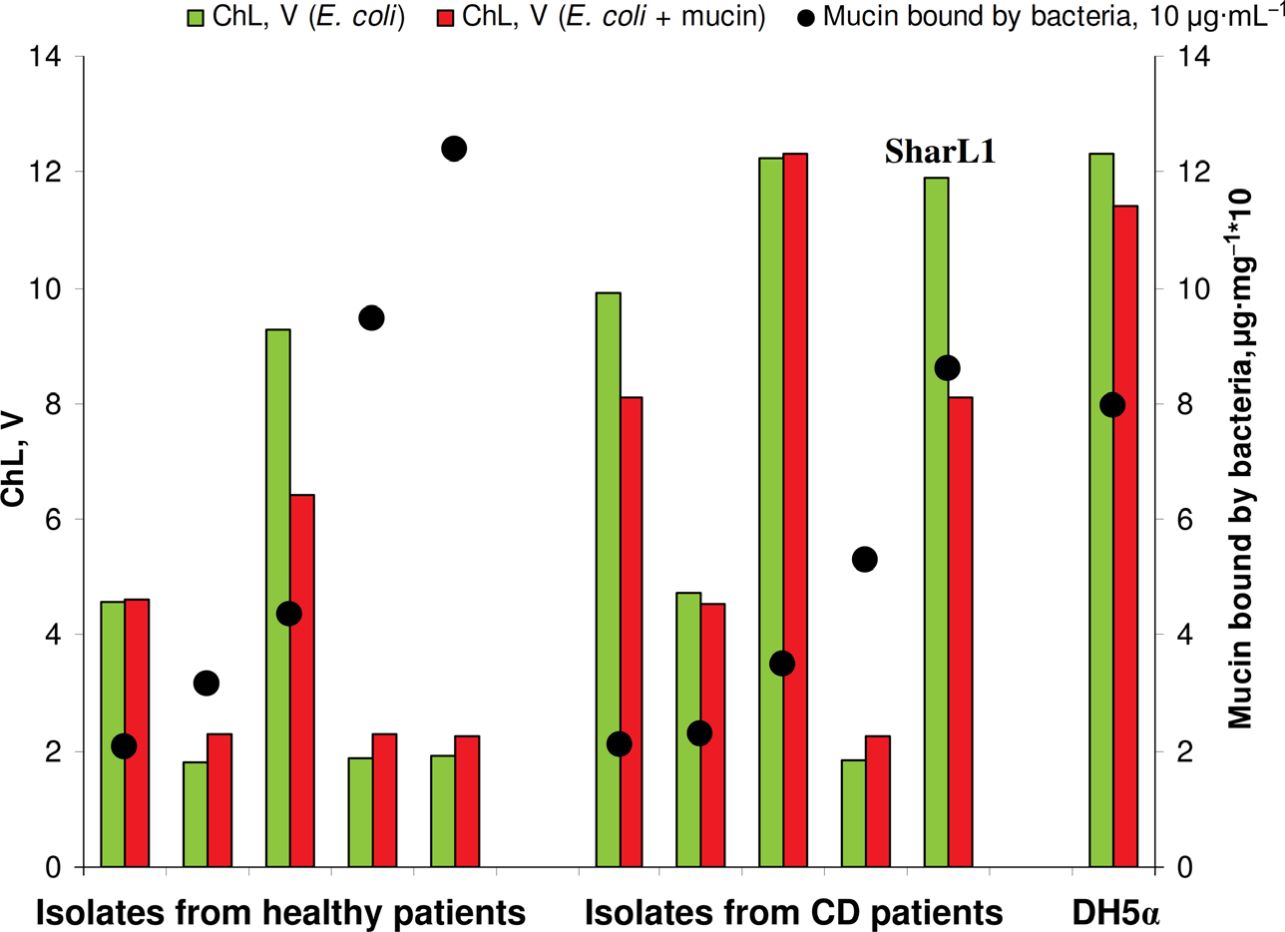
Mucin adsorption and induction of ROS production of blood cells induced by clinical isolates and the laboratory *Escherichia coli* strain, measured in the same experiment. Chemiluminescence is given in light output volts (V).

Neutrophil activation in whole blood without leucocyte isolation was detected in a luminometer cuvette by registering the ROS production via chemiluminescence kinetics and maximum values (ChL). Activation with mucin‐treated and mucin‐untreated bacteria was used to screen 10 clinical *E. coli* isolates and one laboratory strain (Fig. 1, File S2). Three clinical isolates (from healthy and inflamed intestines) demonstrated a curious phenomenon – after treatment with mucin, they demonstrated a lower ability to induce neutrophil activation in blood.

**Figure 2:**
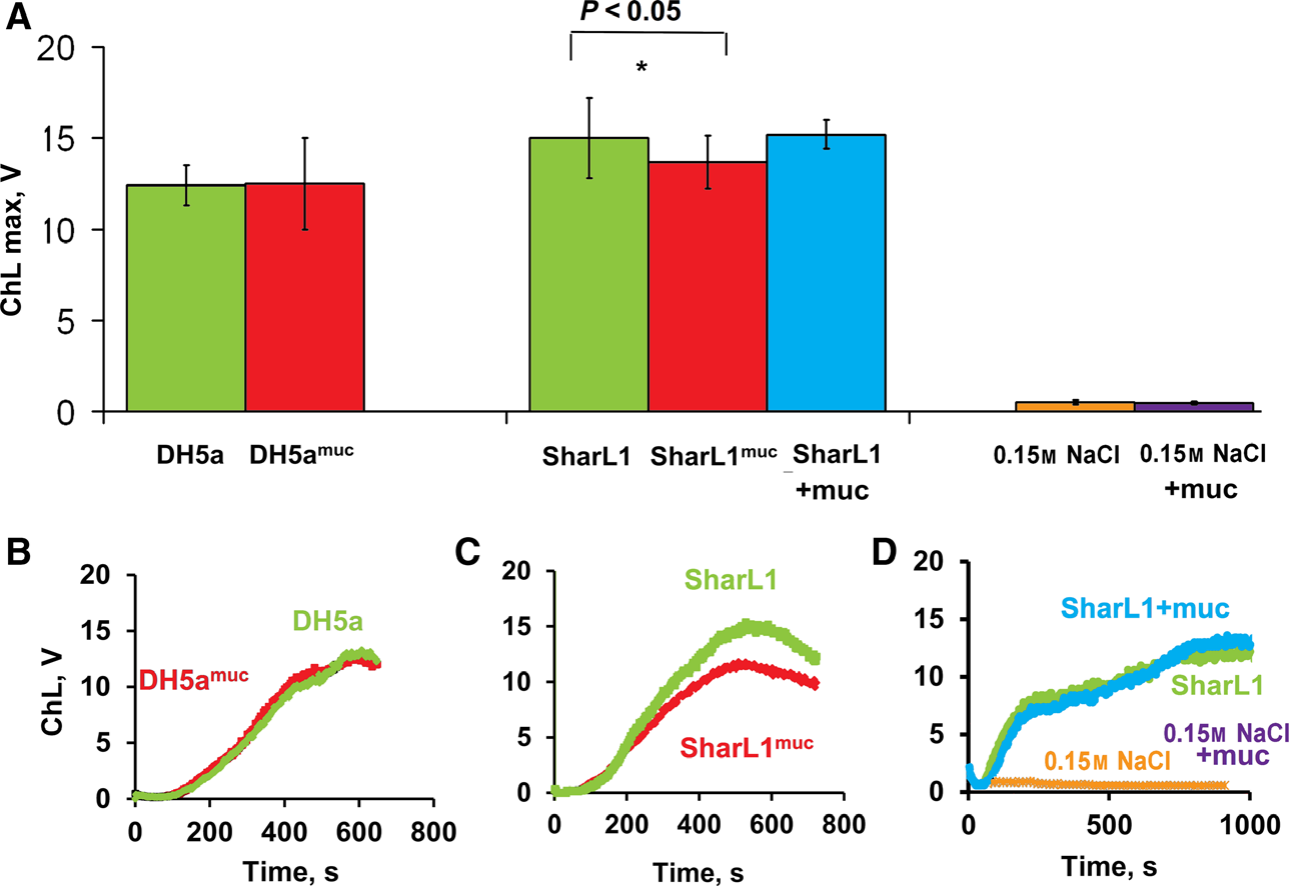
Effect of bacteria‐adsorbed mucin on the ROS production of blood cells induced by SharL1 and DH5α. (A) Peak values for the ChL response of the blood samples (*n* = 3) stimulated with bacteria treated with mucin and the untreated samples. *Significant difference between ChL stimulated by SharL1 and SharL1^muc^ (*P* < 0.05, paired *t*‐test). (B) Typical kinetic curves of the ChL response stimulated by DH5α and DH5α^muc^, (C) kinetics of SharL1 and SharL1^muc^, and (D) SharL1 in the presence (SharL1 + muc) or absence of diluted mucin (30 µg·mL^−1^) and control curves for 0.15 NaCl and 0.15 NaCl + mucin. Chemiluminescence is given in light output volts (V). The error bars represent SD.

For a detailed comparison, two isolates with a similar ability to adsorb mucin (0.08 ± 0.02 mg of mucin per 1 mg DW) were selected including a SharL1 clinical isolate and the laboratory DH5α strain. In a repeated experiment (Fig. 2), SharL1^muc^ (SharL1 cells with adsorbed mucin) induced a significantly lower ChL response than SharL1 (Fig. 2A), though the curve shape and time of peak achievement were similar for both samples (Fig. 2C). At the same time, DH5α and DH5α^muc^ (DH5α cells with adsorbed mucin) did not demonstrate significant differences in the maximum ChL value (Fig. 2A,B). Since the difference in ChL reduction was not due to the amount of adsorbed mucin, this phenomenon was further investigated in experiments using SharL1^muc^ and SharL1.

The direct effect of mucin on leucocytes and the ROS production should have been excluded. The mucin solution (30 µg·mL^−1^) or an equal amount of 0.15 m NaCl were added to the blood cells in a cuvette. After that, the ChL response was stimulated by SharL1 (Fig. 2D). No significant effects by the mucin solution were detected (the difference was 9 ± 23%).

The desialylated mucin was adsorbed by bacteria with the same efficiency, but this adsorption had no effect on ChL stimulation (Table 2).

**Table 2:** Native and desialylated mucin: adsorption by SharL1 cells and effects of adsorbed mucin on the blood ChL peak value (ChLmax)

Parameter	Native mucin	Desialylated mucin
Adsorption, mg·mg^−1^ of bacterial DW	0.089 ± 0.013	0.093 ± 0.020
ChLmax (SharL1muc/SharL1), %	81 ± 8*	99 ± 34

* *P* < 0.05 for SharL1 vs SharL1^muc^ (*t*‐test).

**Figure 3:**
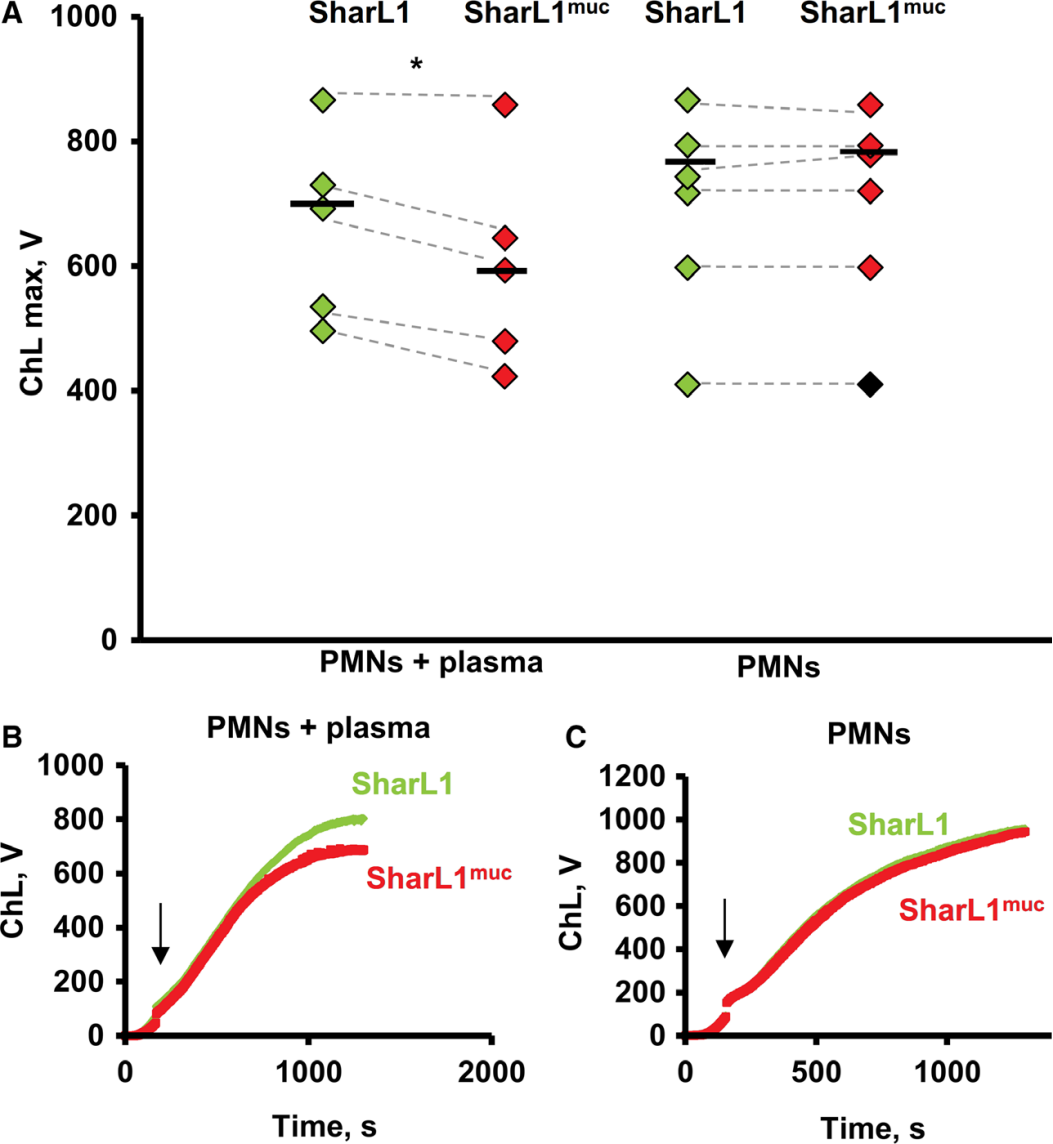
ChL response of isolated neutrophils in the presence of plasma and without plasma stimulated by SharL1 and SharL1^muc^. Peak values for ChL are shown as white diamonds (SharL1) and black diamonds (SharL1^muc^). Values for the same neutrophil sample are connected with the dotted lines (four experiments were performed with blood from three healthy donors). Medians for SharL1 and SharL1^muc^ are indicated with horizontal bars (A). Typical kinetics of the ChL response of the isolated neutrophils with (B) and without (C) plasma after stimulation with SharL1 (grey) and SharL1^muc^ (black). Arrows show the time point when the bacteria were added. *Significant difference between the ChL stimulated by SharL1 and SharL1^muc^ (*P* < 0.05, paired *t*‐test). Maximum values of ChL stimulated by 0.15 NaCl and 0.15 NaCl + mucin were 4.23 + 0.78 and 5.03 + 1.27, correspondingly (those values did not depend on plasma addition to isolated neutrophils).

The ChL response of blood cells was mainly the result of ROS generation by neutrophils 21. When the isolated neutrophils were activated with SharL1 suspension, the ChL response had a greater duration and intensity than in whole blood (Fig. 3A–C). The effect of adsorbed mucin was detectable only in the presence of plasma; the peak ChL value stimulated by SharL1^muc^ was 89.5 ± 5.6% of the ChL induced by SharL1 (*P* < 0.05, paired *t*‐test). At the same time, when isolated neutrophil stimulation was performed without plasma, no difference between SharL1 and SharL1^muc^ was observed and ChL induced by SharL1^muc^ was 101 ± 2.5% of ChL induced by SharL1 (Fig. 3A–C).

### The effects of whole blood incubation with bacteria

Several parameters of neutrophil activation in blood were analysed at two time points on the ChL response curve, including at the area of linear growth (5 min) and at the plateau reached at the highest ChL value (20 min). The blood samples were incubated with SharL1 and SharL1^muc^ as well as with the mucin solution and 0.15 m NaCl, and a complex of parameters was measured with each probe, including the initial blood ChL levels, neutrophil morphological features, MPO and LF in blood plasma and plasma proteins.

#### ROS production by blood cells

**Figure 4:**
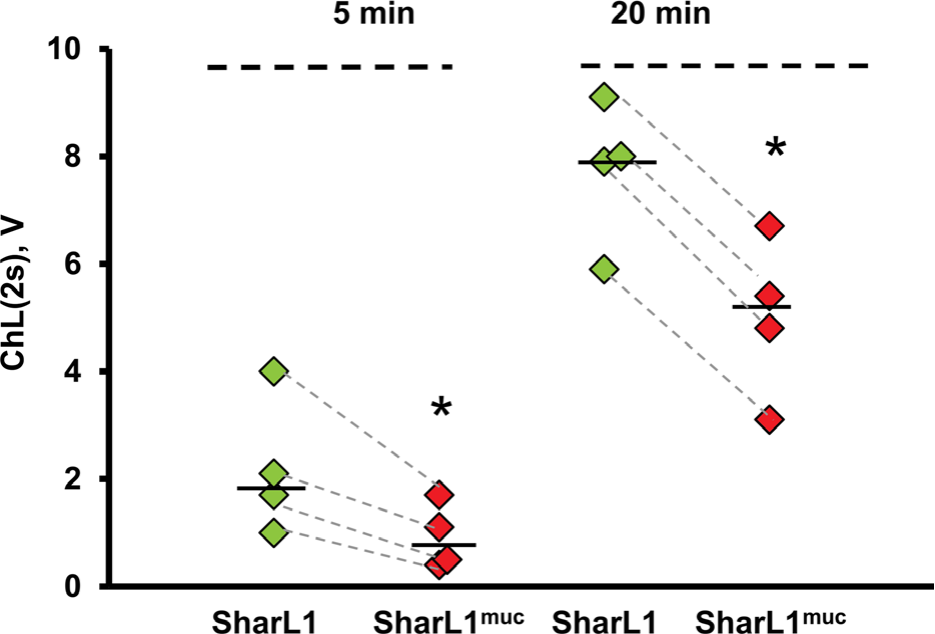
ChL response registered in the blood samples (*n* = 4) after 5 or 20 min of co‐incubation with SharL1 or SharL1^muc^. The ChL response was measured 2 s after the addition of the blood samples with bacteria to the cuvette. Values for the same blood sample are connected with dotted lines. Median values for SharL1 (white diamonds) and SharL1^muc^ (grey diamonds) are indicated with a horizontal line. *P < 0.05 (paired *t*‐test) for SharL1^muc^ vs SharL1. *Significant difference between ChL stimulated by SharL1 and SharL1^muc^ *P* < 0.05 (paired *t*‐test). Maximum values of ChL stimulated by 0.15 NaCl and 0.15 NaCl + mucin were 0.47 + 0.09 and 0.48 + 0.13, correspondingly.

After the incubation of blood with SharL1 or SharL1^muc^ for 5 or 20 min, 20 µL aliquots were immediately added to the luminometer cuvettes and ChL was registered within 2 s. (Fig. 4). The ChL values after 20 min were increased compared with 5 min of incubation, but the difference between SharL1 and SharL1^muc^ was significant (SharL1^muc^ values lower) at both time points.

#### Morphological analysis of neutrophils in the blood

**Figure 5:**
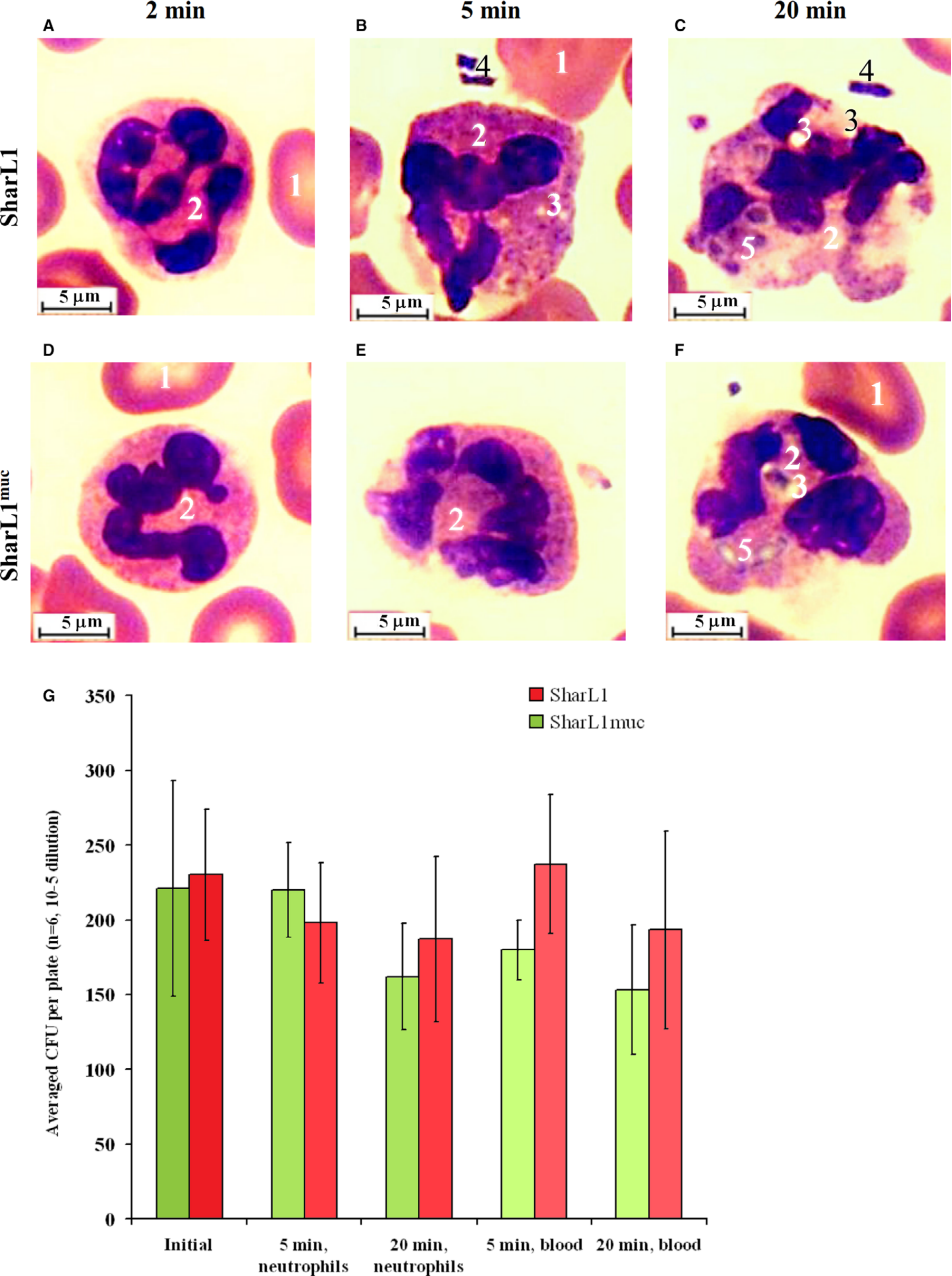
Light microscope images of neutrophils in blood incubated with SharL1 (A–C) or SharL1^muc^ (D–F) for 2 min (A, D), 5 min (B, E) or 20 min (C, F). 1 – erythrocytes, 2 – neutrophils, 3 – vacuoles in the cytoplasm, 4 – extracellular bacteria and 5 – phagocytized and partially lysed bacteria. The length of the scale bars is 5 µm. (G) Effect of mucin adsorption on CFUs of survived bacteria after incubation with neutrophils or blood. The error bars represent SD.

The smears were prepared after incubation of the blood samples with SharL1 or SharL1^muc^ for 2, 5 and 20 min. The images are presented in Fig. 5 and the results in Table 3. There were no morphological reactions in neutrophils after 2 min of incubation (Fig. 5A,D). After 5 min, some vacuoles could be seen in the neutrophil cytoplasm as a marker of cell activation (Fig. 5B,E). At this time point (5 min), the percentage of the activated neutrophils and the number of the vacuoles per cell was lower in the samples with SharL1^muc^ than those with SharL1 (Table 3).

**Table 3:** Morphological activation markers and phagocytosis indexes in neutrophils after incubation of whole blood with SharL1 or SharL1^muc^ for 2, 5 and 20 min

Parameter	SharL1^muc^		SharL1	
	5 min	20 min	5 min	20 min
Neutrophils with vacuole(s), per cent (average ± SD)	58 ± 11*	90 ± 2	86 ± 7*	94 ± 2
Vacuoles/lysosomes, number per cell [min–max (median)]	1–5 (2)	1–5 (3)	1–9 (2)	1–7 (3)
Phagocytized bacteria, number per cell [min–max (median)]	0	1–13 (4)	0	1–7 (3)

* *P* < 0.05 (*t*‐test).

After 20 min of incubation, the neutrophils' structure exhibited various signs of activation. The cell contours became irregular, the nuclei swelled and looked deformed, and there were vacuoles in the major parts of the neutrophils. Moreover, phagocytized bacteria were observed, including those that were partially lysed (Fig. 5C,F). No difference was detected between the blood samples with SharL1 and SharL1^muc^ (Table 3), except that samples with SharL1^muc^ showed 12% neutrophils with 7 or more phagocytized bacteria while samples with SharL1 only showed 3% of such neutrophils.

Further analyses of the blood interactions with bacteria were performed after 5 min of incubation, as the differences between SharL1^muc^ than with SharL1 were observed at this point.

#### Bacteria survival after incubation with isolated neutrophils and whole blood

Bacteria survival assay was performed as follows: 100 µL of bacterial cell suspension (OD600 = 0.2) with or without adsorbed mucin was added to 1 mL of isolated neutrophils or 200 µL of whole blood. After 5 or 20 min of incubation at 25 °C, 50 µL aliquots were sampled, diluted 10^−5^, 10^−6^ and 10^−7^ and plated onto LB agar (50 µL of diluted suspension per plate) for overnight incubation at 37 °C and CFU counting.

When bacterial cells were incubated with isolated neutrophils (no plasma proteins added), mucin adsorption resulted in less survived CFUs at 5 min of incubation and more survived CFUs at 20 min (Fig. 5G). When the whole blood was used for incubation, the increased bacteria survival with adsorbed mucin was observed at both incubation times. CFUs of survived bacteria after 20 min of incubation with neutrophils or blood were always lower than after 5 min of incubation.

#### Determination of plasma myeloperoxidase and lactoferrin concentrations

**Figure 6:**
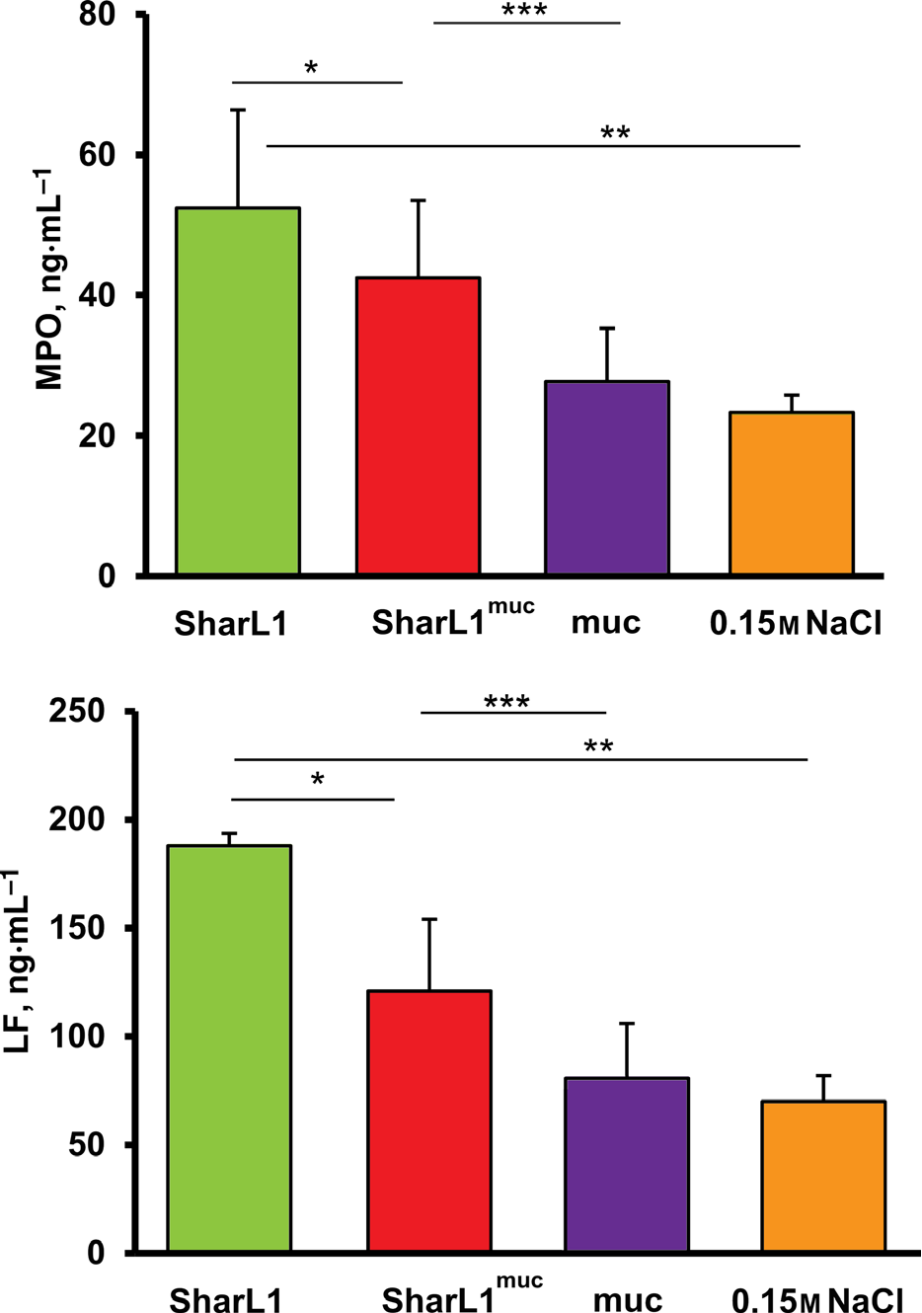
Myeloperoxidase and LF concentration in plasma from blood samples [assays were performed with the blood of five healthy donors (LF) or 4 (MPO)] after 5 min of incubation of blood with SharL1^muc^, SharL1, mucin (20 µg·mL^−1^) or 0.15 m NaCl. The group comparison was performed by pairwise comparison of values achieved for each donor. Asterisks indicate significant differences according to the *t*‐test. *P < 0.05 SharL1^muc^ vs SharL1; **P < 0.05 SharL1 vs 0.15 m NaCl; and ***P < 0.05 SharL1^muc^ vs muc. The error bars represent SD.

The exocytosis of LF and MPO by the activated neutrophils was evaluated by determining the LF and MPO plasma concentration after incubation of the blood with bacteria (SharL1 and SharL1^muc^) for 5 min. The MPO and LF plasma concentrations were increased in plasma from blood incubated with bacteria compared with blood mixed with mucin solution or 0.15 m NaCl (Fig. 6). The mucin solution itself induced no differences in the MPO or LF concentrations compared with 0.15 m NaCl. At the same time, the MPO and LF concentrations after incubation of blood with SharL1^muc^ were significantly lower than with SharL1.

#### HPLC‐MS/MS evaluation of plasma proteins

The effect of mucin adsorption by SharL1 on isolated neutrophil activation was observed only in the presence of plasma. Therefore, plasma proteins were analysed after incubation of blood with SharL1^muc^, SharL1, mucin (20 µg·mL^−1^) and 0.15 m NaCl for 5 min.

**Figure 7:**
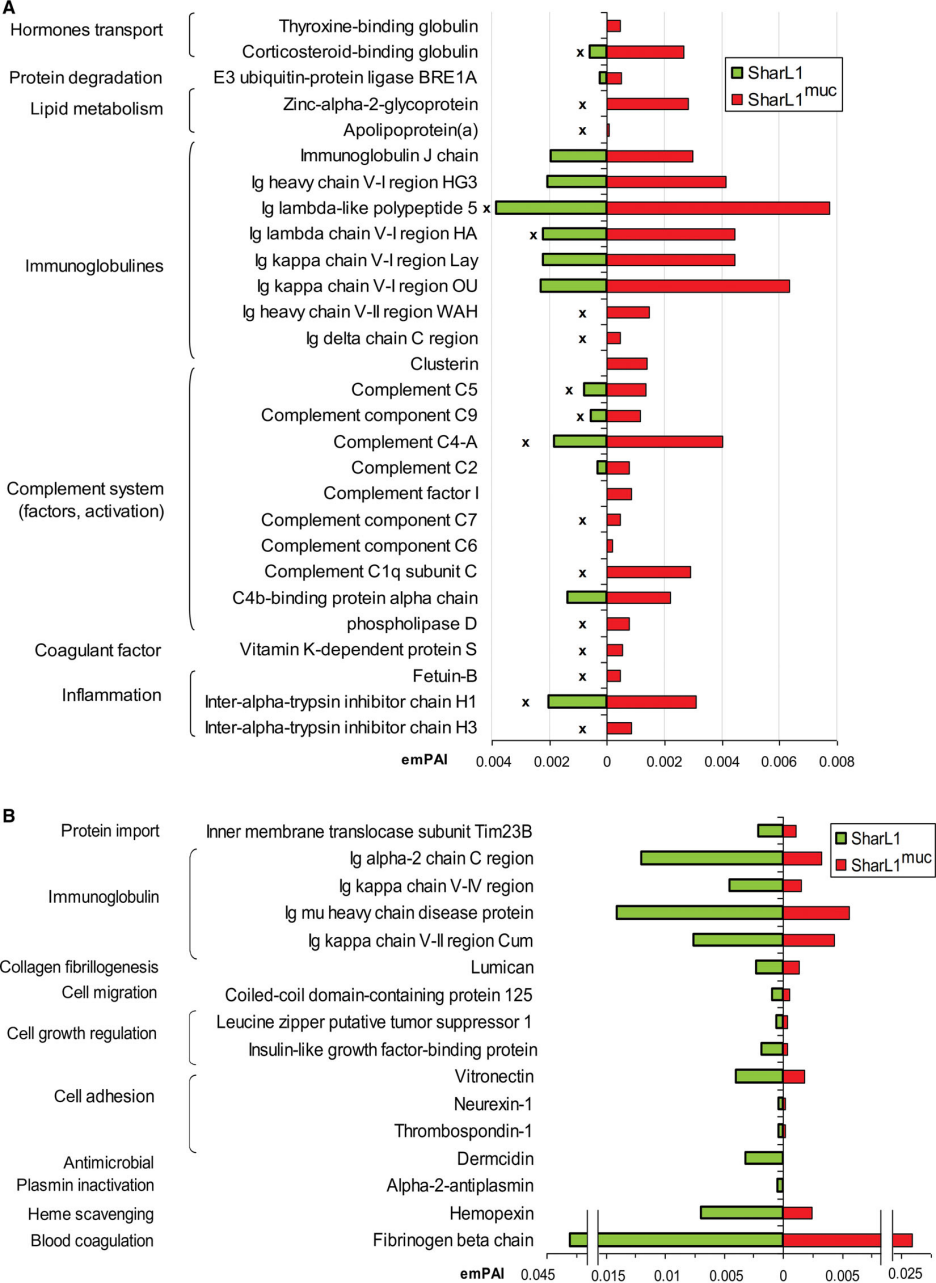
The difference in plasma proteins detected by HPLC‐MS/MS after incubation of blood with SharL1^muc^ and SharL1. Protein relative abundances (emPAI coefficients) are shown. The emPAI of each protein was averaged for two biological repeats and normalized to the total emPAI of the sample. Protein was included if the averaged normalized emPAI in SharL1^muc^ and SharL1 differed by more than 1.5‐fold and no effect was demonstrated by the mucin vs 0.15 m NaCl comparison (File S3). Protein functions were determined according to the UniProt database. ‘x’ indicates proteins which contents in the SharL1 plasma samples were the lowest (compared to plasma from blood incubated with the mucin solution or 0.15 mm NaCl). (A) Proteins that were more abundant in samples with SharL1^muc^ and (B) proteins that were more abundant in samples with SharL1.

More than 100 proteins per plasma sample were identified (two or more identified unique peptides with a score above the cut‐off). File S3 summarizes the search results and links to the deposited raw data. Protein abundances in different samples were compared using coefficient ratios (normalized by the summary emPAI for the sample). Figure 7 shows proteins that were differentially present in plasma after incubation of blood with SharL1^muc^ and SharL1. A protein was included if the averaged normalized emPAI in the plasma‐SharL1^muc^ and plasma‐SharL1 samples differed by more than 1.5‐fold and no effect on plasma proteins was demonstrated by the mucin vs 0.15 m NaCl comparison (File S3).

Bacterial treatment with mucin resulted in an increase of 28 proteins in the plasma (Fig. 7A). Nine of them belong to the complement system and eight to immunoglobulins. For most of these proteins, there was a decrease in plasma‐SharL1 compared with the other plasma samples. This suggests that SharL1 cells partially lose the ability to interact with IgG, complement system proteins and some others (involved in acute inflammation induction) after mucin treatment.

Only 16 proteins were observed as more abundant in plasma‐SharL1 including those involved in blood coagulation (fibrinogen and haemopexin) and cell adhesion as well as four immunoglobulins (Fig. 7B).

### Identification of bacterial membrane proteins that bind mucin *in vitro*

**Figure 8:**
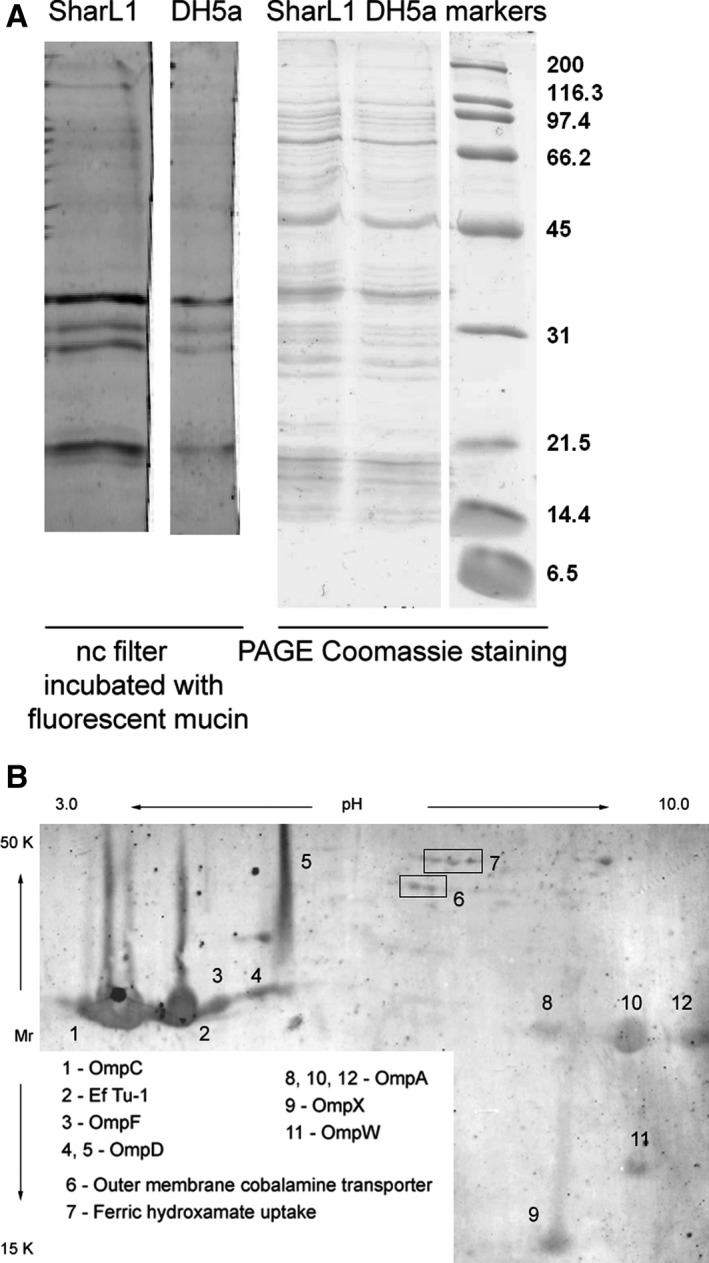
Mucin‐adsorbing membrane proteins from the SharL1 isolate. (A) Mucin‐adsorbing membrane proteins from SharL1 and DH5α. The left panel represents a fluorescent image of a nitrocellulose filter with transferred proteins after separation by PAGE and incubation with a fluorescent‐labelled mucin. The right panel shows proteins separated by 10% SDS/PAGE and Coomassie‐stained. (B) Fluorescent image of a filter with 2D‐PAGE‐separated proteins after incubation with a fluorescent‐labelled mucin. The identified protein spots are indicated. Protein identification details are provided in File S4. Comparison of total bacterial proteome and membrane fraction protein composition is shown in Files S5 and S6

The proteins from the isolated membrane fractions of the SharL1 isolate and DH5α strain were separated by 10% SDS/PAGE and transferred onto a Hybond‐P filter. The filter was then incubated with fluorescent‐labelled mucin (Fig. 8A). The SharL1 isolate and DH5α strain demonstrated similar patterns of mucin‐adsorbing membrane proteins, including three distinct bands with molecular weights of approximately 31K and 21K. To achieve better protein separation for mass‐spectrometry identification, the proteins from the SharL1 membrane fraction were separated by 2D‐PAGE and transferred to a Hybond‐P filter. After incubation with the fluorescent‐labelled mucin, the protein spots were excised and identified by HPLC‐MS/MS mass spectrometry. From the 13 gel fragments, 9 proteins were identified as mucin‐adsorbing (the major protein in each spot was identified as having at least 2 unique peptides and a maximum emPAI coefficient). Six of them were outer membrane proteins (OmpA, OmpC, OmpD, OmpF, OmpX and OmpW). Others included a cobalamine transporter and a ferric hydroxamate uptake protein. One of the most prominent binding spots was identified as the elongation translation factor Ef Tu‐1 (Fig. 8B).

## Discussion

Many reports have suggested that bacteria can exploit mucin secreted by the human mucosa to survive and escape host defence systems 30, 31, 32. It is well known that a lethal dose of various bacteria in animal experimental models can be reduced when mucin is added to bacteria 30. It has also been shown that a 3% mucin solution from porcine stomach was enough to increase the virulence of *E. coli*, *Klebsiella pneumoniae,* *Pseudomonas aeruginosa* and *Acinetobacter baumannii* in a mouse peritonitis model 31. *Salmonella enterica* serovar Typhi enhanced its virulence in a murine model of infection 32. Thus, one can suppose that mucin influences innate immune system activation, and *in vitro* model studies could help to elucidate the mechanism.

In the experiments with the *E. coli* isolate (SharL1) *in vitro,* we have shown that adsorbed mucin downregulated activation of neutrophils in blood (the effect was not common for isolates from Crohn's disease). This is unlikely an artefact of direct neutrophil inhibition by mucin, as mucin in solution had no effect on blood cell activation. This suggested that mucin works only on the bacterial surface. At the same time, adsorbed mucin had no influence on blood cell activation by DH5α cells, suggesting strain‐specific effects.

The fact that the desialylated mucin adsorbed by bacteria did not reduce blood leucocyte activation suggests the participation of mucin sialic acids in this process. Indeed, mechanisms by which sialic acids inhibit neutrophil activation (including oxidative burst) have been described. Several bacteria were reported to exploit Siglec‐9, a human neutrophil lectin that negatively regulates inflammatory responses by recognizing host sialoglycans 33, 34. The sialoglycans used by bacteria for this molecular mimicry could be of bacterial (bacterial sialylated capsular polysaccharide from group B *Streptococcus* 33) or human (sialoglycoproteins adsorbed from host serum by *P. aeruginosa* 35) origin.

Another hypothesis is that adsorbed mucin shielded some structures on the bacterial outer membrane that could be ligands for neutrophil receptors. The most likely mechanism is the inhibition of foreign body opsonization. This is supported by the observation that the effects of adsorbed mucin depended on plasma. As has been shown by other researchers 13, 36, plasma complement proteins participate in the interactions between neutrophils, mucin and bacteria 36 or synthetic polymer surface 13.

We can be sure that our experimental conditions were suitable for opsonization to occur. It was reported that the opsonization of *E. coli* with 10‐20% plasma at 37 °C took 15 min to occur, while 1% serum was minimally opsonic 37, 38. For other bacteria (*Staphylococcus aureus*), 2–5% serum was reported to require 5 min for opsonization to occur 39. In the current study, the co‐incubation of whole blood with bacteria to evaluate ROS generation, neutrophil morphology and plasma proteins was performed with 80% blood at 37 °C for 5 or 20 min. Less blood dilution would lead to even faster opsonization. When ChL kinetics were studied, the final dilution was 4% for blood and 2% for blood plasma and the incubation temperature and time were 37 °C and 10–15 min, respectively. Thus, we can suppose that neutrophil activation and bacterial opsonization proceeded simultaneously in the luminometer cuvette.

The results of the HPLC‐MS/MS analysis of plasma proteins after the incubation with bacteria suggest the ability of the adsorbed mucin to inhibit both immunoglobulin binding and complement activation. Therefore, the opsonization inhibition mechanism was supported even more.

One can speculate about where the interaction between the mucin‐shielded bacteria and neutrophils takes place. Although the SharL1 isolate demonstrated adhesive‐invasive abilities, we suggest that this place is the intestinal lumen. Various studies reported that all the components are present in the intestinal lumen, including the bacteria, mucin monomers 2, recruited neutrophils 9, 10 and secreted immunoglobulins 40 and complement proteins 41, 42, 43.

All *E. coli* proteins that were identified by HPLC‐MS/MS analysis as mucin‐adsorbing *in vitro*, in addition to their various functions, were reported as opsonization targets, such as antigens and immunogenic determinants.

The most prominent among those proteins were the porins that promote passive transport for low‐weight molecules. They also play important roles in adhesion to host cells during infection, virulence, resistance to antibiotics, etc. The same proteins induce nonspecific immunity response; for example, OmpC and OmpF in *E. coli* and OmpD in *S. enterica* are important antigens and vaccine targets 44, 45.

High immunogenicity during infection was reported for the mucin‐binding potential adhesin OmpX, membrane assembly factor OmpW, cobalamine transporter and iron uptake protein 46.

Elongation factor Tu‐1 (EF Tu‐1) is a major bacterial cytoplasmic protein; therefore, its presence in the membrane fraction could be explained by contamination. However, it was reported to be exposed on the cell surface of *Streptococcus, Neisseria* and *Mycoplasma* 47, 48, 49 and under certain conditions on *E. coli* 50. Moreover, it could cause immune response during *Burkholderia* bacterial infection 51 and participate in mucin adsorption by *Lactobacillus* 52, 53.

The experiments with the fluorescent‐labelled mucin suggested that mucin‐adsorbing membrane proteins are similar for the SharL1 isolate and DH5α. The identified proteins are present in both strains and are universal for *E. coli*. Thus, the specific effect of mucin for SharL1 in our study could be due to the differences in protein sequence or posttranslational modifications.

Our findings suggest that mucin was potentially adsorbed by the *E. coli* cell surface via some major outer membrane proteins, resulting in reduced neutrophil activation by the bacteria. It is possible that this was due to the deceleration of bacterial opsonization by the shielding of immunogenic protein epitopes.

## Conflict of interest

The authors declare no conflict of interest.

## Author contributions

EM, NB, TV, DR and OP conceived and designed the project. EM, TV, AS, JB, DR, PS, SG, AAG, ZK, OB and OP acquired the data. EM, DR and OP analysed and interpreted the data. EM and DR wrote the paper.

## Supporting information


**File S1.** PCR reaction products for *tsp*, *yja* and *chuA* genes described in [18] separated by agarose gel electrophoresis. The same result was achieved for 10 separate colonies from the SharL1 isolate.

**File S2.** *E. coli* clinical isolates used in the study, the patient's disease parameters, and mucin–adsorption and its influence on blood activation.

**File S3.** Results of HPLC‐MS/MS identification and nonlabelled emPAI abundance evaluation for plasma proteins after the incubation of blood with SharL1, SharL1^muc^, mucin and 0.15 NaCl. For each type of sample, two biological repeats were analysed. Data, including the samples list, maxis raw files, compound lists produced by Compass Data analysis and search results, are deposited to the ProteomeXchange Consortium (http://proteomecentral.proteomexchange.org) via the PRIDE partner repository 54 with the dataset identifier PXD010496.

**File S4.** Results of HPLC‐MS/MS identification of 2D‐PAGE‐separated SharL1 membrane proteins after transfer to membrane and adsorbing fluorescent‐labelled mucin. Fluorescence intensity of mucin‐binding protein spots measured by ImageQuant TL (Healthcare Life Sciences) is given on a separate sheet. Data, including the samples list, maxis raw files, compound lists produced by Compass Data analysis and search results, are deposited to the ProteomeXchange Consortium (http://proteomecentral.proteomexchange.org) via the PRIDE partner repository 54 with the dataset identifier PXD010472.

**File S5.** 2D PAGE separation of SharL1 total bacterial cell proteome and the membrane proteins fraction. Outer membrane proteins (OMPs) localization is indicated and enriched on the 2D‐PAGE of isolated membrane fraction proteins.

**File S6.** Results of HPLC‐MS/MS identification of proteins from the total bacterial proteome and proteins of SharL1 membrane enriched fraction.

## Data Availability

The HPLC‐MS/MS identification and nonlabelled emPAI abundance evaluation data for plasma proteins after incubation of blood with SharL1, SharL1muc, mucin and 0.15 NaCl, including the sample lists, maxis raw files, compound lists produced by Compass Data analysis and search results, are deposited to the ProteomeXchange Consortium (http://proteomecentral.proteomexchange.org) via the PRIDE partner repository 54 with the dataset identifier PXD010496. Data for the HPLC‐MS/MS identification of *E. coli* SharL1 mucin‐adsorbing membrane proteins, including the sample list, maxis raw files, compound lists produced by Compass Data analysis and search results, are deposited to the ProteomeXchange Consortium (http://proteomecentral.proteomexchange.org) via the PRIDE partner repository 54 with the dataset identifier PXD010472.

## References

[1] Palmela C , Chevarin C , Xu Z , Torres J , Sevrin G , Hirten R , Barnich N , Ng SC and Colombel JF (2018) Adherent‐invasive *Escherichia coli* in inflammatory bowel disease. Gut 67, 574–587. 29141957 10.1136/gutjnl-2017-314903

[2] McGuckin MA , Lindén SK , Sutton P and Florin TH (2011) Mucin dynamics and enteric pathogens. Nat Rev Microbiol 9, 265–278. 21407243 10.1038/nrmicro2538

[3] Ruiz‐Perez F , Wahid R , Faherty CS , Kolappaswamy K , Rodriguez L , Santiago A , Murphy E , Cross A , Sztein MB and Nataro JP (2011) Serine protease autotransporters from Shigella flexneri and pathogenic *Escherichia coli* target a broad range of leukocyte glycoproteins. Proc Natl Acad Sci USA 108, 12881–12886. 21768350 10.1073/pnas.1101006108 PMC3150873

[4] Sajjan SU and Forstner JF (1990) Characteristics of binding of *Escherichia coli* Serotype O157:H7 strain CL‐49 to purified intestinal mucin. Infect Immunity 58, 860–867. 1969394 10.1128/iai.58.4.860-867.1990 PMC258552

[5] Al‐Saedi F , Vaz DP , Stones DH and Krachler AM (2017) 3‐Sulfogalactosyl‐dependent adhesion of *Escherichia coli* HS multivalent adhesion molecule is attenuated by sulfatase activity. J Biol Chem 292, 9792–19803. 10.1074/jbc.M117.817908 PMC5712619 28982977

[6] Vieira MAM , Gomes TAT , Ferreira AJP , Knöbl T , Servin AL and Liévin‐Le Moal V (2010) Two atypical enteropathogenic *Escherichia coli* strains induce the production of secreted and membrane‐bound mucins to benefit their own growth at the apical surface of human mucin‐secreting intestinal HT29‐MTX cells. Infect Immun 78, 927–938. 20065027 10.1128/IAI.01115-09 PMC2825950

[7] Erdem AL , Avelino F , Xicohtencatl‐Cortes J and Girón JA (2007) Host protein binding and adhesive properties of H6 and H7 flagella of attaching and effacing *Escherichia coli* . J Bacteriol 189, 7426–7435. 17693516 10.1128/JB.00464-07 PMC2168434

[8] Pelaseyed T , Bergström JH , Gustafsson JK , Ermund A , Birchenough GM , Schütte A , van der Post S , Svensson F , Rodríguez‐Piñeiro AM , Nyström EE *et al* (2014) The mucus and mucins of the goblet cells and enterocytes provide the first defense line of the gastrointestinal tract and interact with the immune system. Immunol Rev 260, 8–20. 24942678 10.1111/imr.12182 PMC4281373

[9] Fournier BM and Parkos CA (2012) The role of neutrophils during intestinal inflammation. Mucosal Immunol 5, 354–366. 22491176 10.1038/mi.2012.24

[10] Loetscher Y , Wieser A , Lengefeld J , Kaiser P , Schubert S , Heikenwalder M , Hardt WD and Stecher B (2012) *Salmonella* transiently reside in luminal neutrophils in the inflamed gut. PLoS ONE 7, e34812. 22493718 10.1371/journal.pone.0034812 PMC3321032

[11] Bansil R and Turner BS (2018) The biology of mucus: composition, synthesis and organization. Adv Drug Deliv Rev 124, 3–15. 28970050 10.1016/j.addr.2017.09.023

[12] McGuckin M , Linden SK , Sutton P and Florin TH (2011) Mucin dynamics and enteric pathogens. Nat Rev Microbiology 9, 265–278. 21407243 10.1038/nrmicro2538

[13] Sandberg T , Carlsson J and Ott MK (2009) Interactions between human neutrophils and mucin‐coated surfaces. J Mater Sci Mater Med 20, 621–631. 18925363 10.1007/s10856-008-3595-y

[14] Aknin M‐LR , Berry M , Dick AD and Khan‐Lim D (2004) Normal but not altered mucins activate neutrophils. Cell Tissue Res 318, 545–551. 15490242 10.1007/s00441-004-0957-8

[15] Vakhrusheva TV , Baikova YuP , Balabushevich NG , Gusev SA , Lomakina GYu , Sholina EA , Moshkovskaya MA , Shcherbakov PL , Pobeguts OV and Mikhalchik EV (2018) Binding of mucin by *E. coli* from human gut. Bulletin Exp Biol Med 165, 235–238. 10.1007/s10517-018-4137-3 29923001

[16] Silverberg MS , Satsangi J , Ahmad T , Arnott ID , Bernstein CN , Brant SR , Caprilli R , Colombel J‐F , Gasche C , Geboes K *et al* (2005) Toward an integrated clinical, molecular and serological classification of inflammatory bowel disease: Report of a Working Party of the 2005 Montreal World Congress of Gastroenterology. Can J Gastroenterol 19(Suppl A), 5–36. 10.1155/2005/269076 16151544

[17] Best WR , Becktel JM , Singleton JW and Kern F Jr (1976) Development of a Crohn's disease activity index. National cooperative Crohn's disease study. Gastroenterology 70, 439–444. 1248701

[18] Clermont O , Bonacorsi S and Bingen E (2000) Rapid and simple determination of the *Escherichia coli* phylogenetic group. Appl Environ Microbiol 66, 4555–4558. 11010916 10.1128/aem.66.10.4555-4558.2000 PMC92342

[19] Turner BS , Bhaskar KR , Hadzopoulou‐Cladaras M and LaMont JT (1999) Cysteine‐rich regions of pig gastric mucin contain vonWillebrand factor and cystine knot domains at the carboxyl terminal. Biochim Biophys Acta‐Gene Struct Expr 1447, 77–92. 10.1016/s0167-4781(99)00099-8 10500247

[20] Balabushevich NG , Sholina EA , Mikhalchik EV , Filatova LY , Vikulina AS and Volodkin D (2018) Self‐assembled mucin‐containing microcarriers via hard templating on CaCO_3_ crystals. Micromachines 9, 307. 10.3390/mi9060307 PMC6187553 30424240

[21] Lindena J , Burkhardt H and Dwenger A (1987) Mechanism of non‐opsonized zymosan‐induced and luminol‐enhanced chemiluminescence in whole blood and isolated phagocytes. J Clin Chem Clin Biochem 25, 765–778. 3440857 10.1515/cclm.1987.25.11.765

[22] Adewoyin AS and Nwogoh B (2014) Peripheral blood film – a review. Ann Ib Postgrad Med 12, 71–79. 25960697 PMC4415389

[23] Kono M , Saigo K , Matsuhiroya S , Takahashi T , Hashimoto M , Obuchi A and Kawano S (2018) Detection of activated neutrophils by reactive oxygen species production using a hematology analyzer. J Immunol Methods 463, 122–126. 30339797 10.1016/j.jim.2018.10.004

[24] Gorudko IV , Tcherkalina OS , Sokolov AV , Pulina MO , Zakharova ET , Vasilyev VB , Cherenkevich SN and Panasenko OM (2009) New approaches to the measurement of the concentration and peroxidase activity of myeloperoxidase in human blood plasma. Bioorg Khim 35, 629–639. 19915640 10.1134/s1068162009050057

[25] Sokolov AV , Zakahrova ET , Kostevich VA , Samygina VR and Vasilyev VB (2014) Lactoferrin, myeloperoxidase, and ceruloplasmin: complementary gearwheels cranking physiological and pathological processes. Biometals 27, 815–828. 24966132 10.1007/s10534-014-9755-2

[26] Fujiki Y , Hubbard AL , Fowler S and Lazarow PB (1982) Isolation of intracellular membranes by means of sodium carbonate treatment: application to endoplasmic reticulum. J Cell Biol 93, 97–102. 7068762 10.1083/jcb.93.1.97 PMC2112113

[27] Matyushkina D , Pobeguts O , Butenko I , Vanyushkina A , Anikanov N , Bukato O , Evsyutina D , Bogomazova A , Lagarkova M , Semashko T *et al* (2016) Phase transition of the bacterium upon invasion of a host cell as a mechanism of adaptation: a *Mycoplasma gallisepticum* model. Sci Rep 24, 35959. 10.1038/srep35959 PMC5075909 27775027

[28] Matyushkina DS , Butenko IO , Pobeguts OV , Fisunov GY and Govorun VM (2017) Proteomic response of bacteria during the interaction with a host cell in a model of *Mycoplasma gallisepticum* . Russ J Bioorg Chem 43, 507–517.

[29] Shinoda K , Tomita M and Ishihama Y (2010) emPAI Calc–for the estimation of protein abundance from large‐scale identification data by liquid chromatography‐tandem mass spectrometry. Bioinformatics 26, 576–577. 20031975 10.1093/bioinformatics/btp700

[30] Nungester W , Wolf A and Jourdonais L (1932) Effect of gastric mucin on virulence of bacteria in intraperitoneal injections in the mouse. Proc Soc Exp Biol Med 30, 120–121.

[31] Park JY , Park C , Chun H‐S , Byun J‐H , Cho S‐Y and Lee D‐G (2017) Establishment of experimental murine peritonitis model with hog gastric mucin for carbapenem‐resistant gram‐negative bacteria. Infect Chemother 49, 57–61. 28271653 10.3947/ic.2017.49.1.57 PMC5382051

[32] Secundino I , López‐Macías C , Cervantes‐Barragán L , Gil‐Cruz C , Ríos‐Sarabia N , Pastelin‐Palacios R , Villasis‐Keever MA , Becker I , Puente JL , Calva E *et al* (2006) *Salmonella* porins induce a sustained, lifelong specific bactericidal antibody memory response. Immunology 117, 59–70. 16423041 10.1111/j.1365-2567.2005.02263.x PMC1782194

[33] Carlin AF , Uchiyama S , Chang YC , Lewis AL , Nizet V and Varki A (2009) Molecular mimicry of host sialylated glycans allows a bacterial pathogen to engage neutrophil Siglec‐9 and dampen the innate immune response. Blood 113, 3333–3336. 19196661 10.1182/blood-2008-11-187302 PMC2665898

[34] Lizcano A , Secundino I , Döhrmann S , Corriden R , Rohena C , Diaz S , Ghost P , Deng L , Nizet V and Varki A (2017) Erythrocyte sialoglycoproteins engage Siglec‐9 on neutrophils to suppress activation. Blood 29, 3100–3110. 10.1182/blood-2016-11-751636 PMC5465837 28416510

[35] Khatua B , Bhattacharya K and Mandal C (2012) Sialoglycoproteins adsorbed by *Pseudomonas aeruginosa* facilitate their survival by impeding neutrophil extracellular trap through siglec‐9. J Leukoc Biol 91, 641–655. 22241833 10.1189/jlb.0511260

[36] Lambert HP and Richley J (1952) The action of mucin in promoting infections: the anticomplementary effect of mucin extracts and certain other substances. Br J Exp Pathol 33, 327–339. 14954104 PMC2073408

[37] Falkenhagen U , Easmon CS , Nimmich W , Zingler G and Naumann G (1986) Stimulation of particle‐induced chemiluminescence in human granulocytes by various *Escherichia coli* strains. Br J Exp Pathol 67, 297–303. 2423108 PMC2013165

[38] Tofte RW , Peterson PK , Kim Y and Quie PG (1980) Influence of serum concentration on opsonization by the classical and alternative complement pathways. Infect Immun 27, 693–696. 6991425 10.1128/iai.27.2.693-696.1980 PMC550821

[39] Verbrugh HA , Van Dijk WC , Peters R , Van Der Tol ME , Peterson PK and Verhoef J (1979) *Staphylococcus aureus* opsonization mediated via the classical and alternative complement pathways. A kinetic study using MgEGTA chelated serum and human sera deficient in IgG and complement factors C1s and C2. Immunology 36, 391–397. 108204 PMC1457553

[40] Macpherson AJ , McCoy KD , Johansen FE and Brandtzaeg P (2008) The immune geography of IgA induction and function. Mucosal Immunol 1, 11–22. 19079156 10.1038/mi.2007.6

[41] Andoh A , Fujiyama Y , Sumiyoshi K and Bamba T (1996) Local secretion of complement C3 in the exocrine pancreas: ductal epithelial cells are possible bio‐synthetic site. Gastroenterology 110, 1919. 8964419 10.1053/gast.1996.v110.pm8964419

[42] Ahrenstedt O , Knutson B , Nisson K , Nilsson‐Ekdahl K , Odlind K and Hallgren R (1990) Enhanced local production of complement components in the small intestines of patients with Crohn's disease. N Engl J Med 322, 1345. 2325733 10.1056/NEJM199005103221903

[43] Andoh A , Fujiyama Y , Sakumoto H , Uchihara H , Kimura T , Koyama S and Bamba T (1998) Detection of complement C3 and factor B gene expression in normal colorectal mucosa, adenoma and carcinoma. Clin Exp Immunol 111, 477. 9528886 10.1046/j.1365-2249.1998.00496.x PMC1904873

[44] Liu C , Chen Z , Tan C , Liu W , Xu Z , Zhou R and Chen H (2012) Immunogenic characterization of outer membrane porins OmpC and OmpF of porcine extraintestinal pathogenic *Escherichia coli* . FEMS Microbiol Lett 337, 104–111. 23003111 10.1111/1574-6968.12013

[45] Gil‐Cruz C , Bobat S , Marshall JL , Kingsley RA , Ross EA , Henderson IR , Leyton DL , Coughlan RE , Khan M , Jensen KT *et al* (2009) The porin OmpD from nontyphoidal *Salmonella* is a key target for a protective B1b cell antibody response. Proc Natl Acad Sci USA 106, 9803–9808. 19487686 10.1073/pnas.0812431106 PMC2701014

[46] Hagan EC and Mobley HLT (2007) Uropathogenic *Escherichia coli* outer membrane antigens expressed during urinary tract infection. Infect Immun 75, 3941–3949. 17517861 10.1128/IAI.00337-07 PMC1951972

[47] Rodriguez‐Ortega MJ , Norais N , Bensi G , Liberatori S , Capo S , Mora M , Scarselli M , Doro F , Ferrari G *et al* (2006) Characterization and identification of vaccine candidate proteins through analysis of the group A *Streptococcus* surface proteome. Nat Biotechnol 24, 191–197. 16415855 10.1038/nbt1179

[48] Williams JN , Skipp PJ , Humphries HE , Christodoulides M , O'Connor CD and Heckels JE (2007) Proteomic analysis of outer membranes and vesicles from wild‐type serogroup B *Neisseria meningitides* and a lipopolysaccharide‐deficient mutant. Infect Immun 75, 1364–1372. 17158897 10.1128/IAI.01424-06 PMC1828559

[49] Widjaja M , Harvey KL , Hagemann L , Berry IJ , Jarocki VM , Raymond BBA , Tacchi JL , Gründel A , Steele JR , Padula MP *et al* (2017) Elongation factor Tu is a multifunctional and processed moonlighting protein. Sci Rep 7, 11227. 28894125 10.1038/s41598-017-10644-z PMC5593925

[50] Dombou M , Bhide SV and Mizushima S (1981) Appearance of elongation factor Tu in the outer membrane of sucrose‐dependent spectinomycin‐resistant mutants of *Escherichia coli* . Eur J Biochem 113, 397–403. 6451426 10.1111/j.1432-1033.1981.tb05079.x

[51] Nieves W , Heang J , Asakrah S , Höner zu Bentrup K , Roy CJ and Morici LA (2010) Immunospecific responses to bacterial elongation factor Tu during *Burkholderia* infection and immunization. PLoS ONE 5, e14361. 21179405 10.1371/journal.pone.0014361 PMC3003680

[52] Granato D , Bergonzelli GE , Pridmore RD , Marvin L , Rouvet M and Corthésy‐Theulaz IE (2004) Cell surface‐associated elongation factor Tu mediates the attachment of *Lactobacillus johnsonii* NCC533 (La1) to human intestinal cells and mucins. Infect Immun 72, 2160–2169. 15039339 10.1128/IAI.72.4.2160-2169.2004 PMC375183

[53] Nishiyama K , Ochiai A , Tsubokawa D , Ishihara K , Yamamoto Y and Mukai T (2017) Correction: identification and characterization of sulfated carbohydrate‐binding protein from *Lactobacillus reuteri* . PLoS ONE 12, e0174257. 28291819 10.1371/journal.pone.0174257 PMC5349683

[54] Vizcaíno JA , Côté RG , Csordas A , Dianes JA , Fabregat A , Foster JM , Griss J , Alpi E , Birim M , Contell J , *et al* (2013) The Proteomics Identifications (PRIDE) database and associated tools: status in 2013. Nucleic Acids Res. 41 (D1), D1063–D1069. 23203882 10.1093/nar/gks1262 PMC3531176

