# Supplementary material for: Mucin adsorbed by E. coli can affect neutrophil activation in vitro
Source: FEBS Open Bio. 2019 Dec 19;10(2):180–96. doi: 10.1002/2211-5463.12770 (PMC6996330; doi:10.1002/2211-5463.12770)
Supplement: Supplementary file 1 — File S1. PCR reaction products for tsp, yja and chuA genes described in [18] separated by agarose gel electrophoresis. The same result was achieved for 10 separate colonies from the SharL1 isolate. [file FEB4-10-180-s001.docx]

**Supplementary File 1. Characteristics of isolate SharL1.**

Patient (male, 26 years old). Diagnosis: Crohn’s disease, ileocolitis, ileum, sigmoid, caecum affected. Lymphofollicular hyperplasia of ileum, ileitis, aphthosic proctosigmoiditis.

Isolation of *E. coli*.

In this study we used laboratory strain DH5α and the clinical isolate, achieved from the liquid ileal content of Crohn’s disease patient during diagnostic endoscopy at Central Scientific Institute of Gastroenterology (Moscow Clinical Research Centre, Moscow). The material collection was approved by the local Ethics Committee, the patient has given written informed consent for research and publication of data. The liquid ileal content was plated onto agar LB medium and incubated at 37^o^C for 16 h. Species of individual colonies were identified by Biotyper system and mass-spectrometer Bruker Microflex (Bruker, Germany). Isolate SharL1, used in this study, belonged to phylogroup B2, demonstrated adhesion (not invasion) to CaCo cells, and mobility.

The isolate was cultivated in LB at 37^o^C (200 RPM) for 14 h. Bacterial cells were harvested by centrifugation (3 500 g, 15 min) and the pellet was washed twice with PBS. Bacterial suspensions used in experiments were normalized by absorbance at 540 nm (OD 540). 1 OD unit in 1 ml cuvette was equal to 0.2 mg/ml of the dry weight of bacteria (DW) and contained 8∙10^8^ CFU/ml.

Phylogroup determination for SharL1 isolate.

Phylogroup determination of SharL1 was determined by PCR-reaction with primers for *tsp*, *yja* and *chuA* genes as described in [1].

Fig. 1 below illustrates that DNA extracted from SharL1 culture used as PCR matrix produced PCR-products equivalent for those of all three genes, indicating that SharL1 isolate belongs to phylogroup B2.


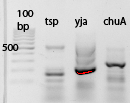


Fig. S1. Agarose gel phoresis of PCR-reaction products for *tsp*, *yja* and *chuA* genes as described in [1]. The same picture was achieved for 10 separate colonies from SharL1 isolate.

[1] Clermont O, Bonacorsi S, and Bingen E. Rapid and Simple Determination of the *Escherichia coli* Phylogenetic Group. Appl Environ Microbiol. 2000 Oct; 66(10): 4555–4558.

The ability of SharL1 isolate for the adhesion to monolayer of CaCo cells.

The isolate was cultivated on LB (37^o^C, 200 RPM) were harvested by centrifugation (3500 g, 15 min) at mid-log phase. Pellet was washed with PBS and resuspended in PBS. 150 ul of bacterial suspension (OD 620 = 0.2) were mixed with 5 ml DMEM + 20% FBS. Bacteria in DMEM (500 ul) were added to CaCo cells, grown in 24 well plate till the formation of the monolayer (about 500 000 CaCo cells per well). Bacteria suspension control 1 (control-1) was collected at this point and plated onto LB-agar plates at 10^-3^, 10^-4^ dilution. Plate with CaCo and bacteria was incubated for 3 h at 37^o^C. After incubation, the monolayer was gently washed twice with PBS to remove unbound bacteria, cells were removed from the plate by trypsin (200 ul), lysed with 200 ul of DMEM (20% FBS) and 200 ul of 1% Triton X100. The solution was mixed and plated on agar LB at 10^-2^, 10^-3^ dilution (5 ul per agar plate).

Colony number per agar plate = CFU per well*100

Adhesion (+ invasion) was determined as [CFU per well / control 1 * 500 000 (number of CaCo cells per well)].

Comparative data for SharL1 isolate and MG1655 laboratory strain are given in Table S1. According to them, SharL1 demonstrates 24 times more effective adhesion to CaCo than MG1655, this it can be defined as adhesive.

Mobility test.

Bacteria mobility was determined by needle struck in the thick layer of 0.4 % LB agar. After 24 h incubation at 37^o^C, the diameter of bacteria spot was measured. SharL1 isolate demonstrated mobility of 17,54±7,47 mm, 5 times higher than Mg1655 (3,46 ±0,26 mm).

Table S1 (values averaged for 3 biological and 3 mechanical repeats)

| ***E. coli* isolate** | **SharL1** | **SharL1** | **MG1655** | **MG1655** |
| --- | --- | --- | --- | --- |
|  | **Median value** | **Stand. dev.** | **Median value** | **Stand. dev.** |
| **control-1, x10^4^** | 12,625 | 3,852179 | 18,875 | 2,531939 |
| **adhesion+invasion, x10^3^** | 30,125 | 4,580627 | 1,9875 | 0,23566 |
| **% of bacteria that adhered (invaded) to CaCo cells** | 2,386139 | 1,1891 | 0,105298 | 0,093075 |
