# Supplementary material for: Mucin adsorbed by E. coli can affect neutrophil activation in vitro
Source: FEBS Open Bio. 2019 Dec 19;10(2):180–96. doi: 10.1002/2211-5463.12770 (PMC6996330; doi:10.1002/2211-5463.12770)
Supplement: Supplementary file 2 — File S2. E. coli clinical isolates used in the study, the patient's disease parameters, and mucin–adsorption and its influence on blood activation. [file FEB4-10-180-s002.docx]

**Supplementary File 2.**

Table S2. The ability of *E. coli* clinical isolates to bind mucin and its effect on neutrophil activation.

|  |  | **CL max, (Lum)** | |  |  |  |  |  |
| --- | --- | --- | --- | --- | --- | --- | --- | --- |
|  | **Patient** | ***E. coli*** | ***E. coli* + mucin** | **mucin bound by bacteria (μg/mg)*10** | **localisation** | **clinical activity** | **endoscopic activity** | **source of *E. coli*** |
| **CD** | A | 9,9 | 8,1 | 2,1 | ileitis | mild disease | 10 | lumen |
|  | B | 4,7 | 4,6 | 2,3 | ileocolitis | moderate disease | 14 | biopsy |
|  | M | 12,2 | 12,3 | 3,5 | ileocolitis | mild disease | 15 | lumen |
|  | V | 1,8 | 2,2 | 5,3 | ileocolitis | mild disease | 13 | lumen |
|  | SharL1 | 11,9 | 8,1 | 8,6 | ileocolitis |  |  | lumen |
| **healthy** | h-1 | 4,6 | 4,6 | 2,1 |  | no IBD |  | lumen |
|  | h-2 | 1,8 | 2,3 | 3,2 |  | no IBD |  | feces |
|  | h-3 | 9,3 | 6,4 | 4,3 |  | no IBD |  | feces |
|  | h-4 | 1,9 | 2,3 | 9,4 |  | no IBD |  | feces |
|  | h-5 | 1,9 | 2,2 | 12,4 |  | no IBD |  | feces |
| **lab strain** | DH5α | 12,3 | 11,4 | 7,9 |  |  |  |  |
