# Supplementary material for: Mucin adsorbed by E. coli can affect neutrophil activation in vitro
Source: FEBS Open Bio. 2019 Dec 19;10(2):180–96. doi: 10.1002/2211-5463.12770 (PMC6996330; doi:10.1002/2211-5463.12770)

Supplementary File 5. 2D PAGE separation of SharL1 total bacterial cell proteome and the membrane proteins fraction. Outer membrane proteins (OMPs) localization is indicated and enriched on the 2D-PAGE of isolated membrane fraction proteins.


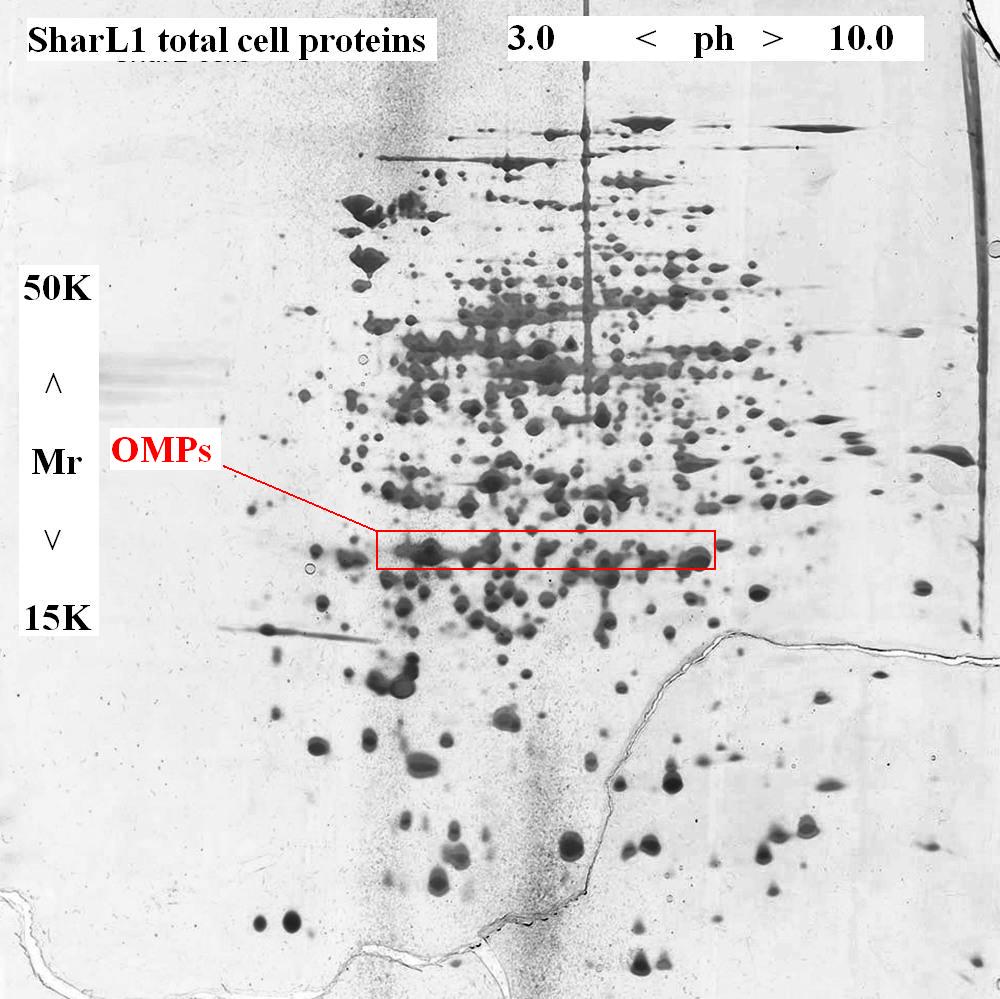


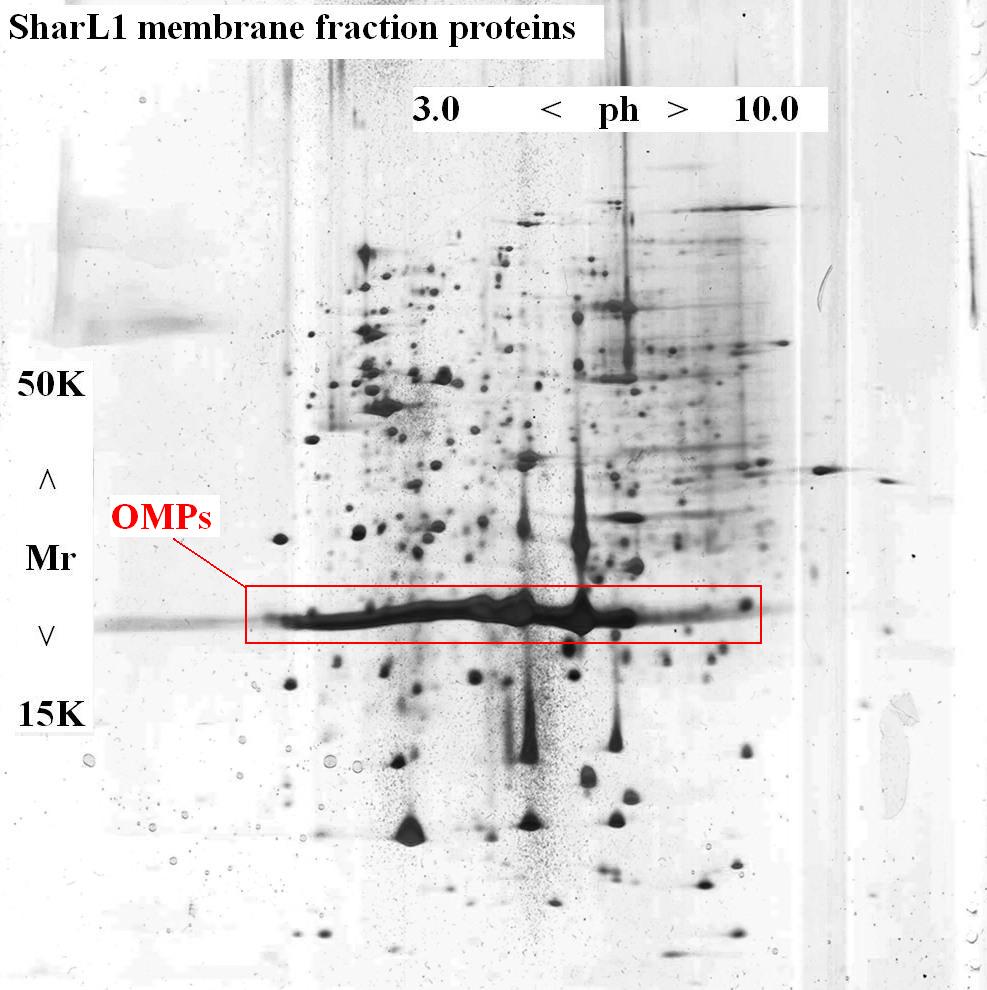

Supplement: Supplementary file 5 — File S5. 2D PAGE separation of SharL1 total bacterial cell proteome and the membrane proteins fraction. Outer membrane proteins (OMPs) localization is indicated and enriched on the 2D‐PAGE of isolated membrane fraction proteins. [file FEB4-10-180-s005.docx]
